# Identification and Functional Characterization of Two Homologous SpoVS Proteins Involved in Sporulation of Bacillus thuringiensis

**DOI:** 10.1128/Spectrum.00881-21

**Published:** 2021-10-06

**Authors:** Xinlu Liu, Ruibin Zhang, Shuo Hou, Huanhuan Liu, Jiaojiao Wang, Qingyue Yu, Qi Peng, Fuping Song

**Affiliations:** a State Key Laboratory for Biology of Plant Diseases and Insect Pests, Institute of Plant Protection, Chinese Academy of Agricultural Sciences, Beijing, China; University of Minnesota

**Keywords:** *Bacillus thuringiensis*, *spoVS*, sporulation, disporic septum, σ^H^

## Abstract

Sporulation is an important part of the life cycle of Bacillus thuringiensis and the basis for the production of parasporal crystals. This study identifies and characterizes two homologous *spoVS* genes (*spoVS1* and *spoVS2*) in B. thuringiensis, both of whose expression is dependent on the σ^H^ factor. The disruption of *spoVS1* and *spoVS2* resulted in defective B. thuringiensis sporulation. Similar to Bacillus subtilis, B. thuringiensis strain HD(Δ*spoVS1*) mutants showed delayed formation of the polar septa, decreased sporulation efficiency, and blocked spore release. Different from B. subtilis, B. thuringiensis HD(Δ*spoVS1*) mutants had disporic septa and failed to complete engulfment in some cells. Moreover, HD(Δ*spoVS2*) mutants had delayed spore release. The effect of *spoVS1* deletion on polar septum delay and sporulation efficiency could be compensated by *spoVS2*. β-Galactosidase activity analysis showed that the expression of pro-*sigE* and *spoIIE* decreased to different degrees in the HD(Δ*spoVS1*) and HD(Δ*spoVS2*) mutants. The different effects of the two mutations on the expression of sporulation genes led to decreases in Cry1Ac production of different levels.

**IMPORTANCE** There is only one *spoVS* gene in B. subtilis, and its effects on sporulation have been reported. In this study, two homologous *spoVS* genes were found and identified in B. thuringiensis. The different effects on sporulation and parasporal crystal protein production in B. thuringiensis and their relationship were investigated. We found that these two homologous *spoVS* genes are highly conserved in the Bacillus cereus group, and therefore, the functional characterization of SpoVS is helpful to better understand the sporulation processes of members of the Bacillus cereus group.

## INTRODUCTION

Sporulation is an important developmental process that enables *Bacillus* cells to resist harsh environments. In Bacillus subtilis, an important bacterial model, it has been confirmed that the sporulation process is divided into seven stages that are mediated by a series of sigma factors: σ^H^, σ^F^, σ^E^, σ^G^, and σ^K^ (1). Various cytological events occur during these seven stages, as follows: stage 0 to I, axial filamentation; stage II, polar septum formation; stage III, forespore engulfment; stage IV to stage V, cortex and coat assembly; and stage VI to VII, spore maturation and mother cell lysis (1–6). Forespore engulfment, mediated by σ^F^ and σ^E^, is a key step in the compartmentalization of cell regions into forespore and mother cells. Septal thinning and membrane migration are important in the process of forespore engulfment. Septal thinning is blocked in *spoIID*, *spoIIM*, and *spoIIP* mutants (7–10), and membrane migration is blocked in the *spoIIB spoVG* double mutant. However, deletion of the *spoVS* gene, controlled by σ^H^, allows the *spoIIB spoVG* double mutant to complete engulfment (11, 12). In addition, *spoVS* mutations delay polar septum development and block sporulation at stage V (11).

The Bacillus cereus group is another important clade of *Bacillus* species. B. anthracis, B. cereus, and B. thuringiensis are the most well-studied members of this group (13). B. anthracis and B. cereus can produce toxins that are pathogenic to human beings (14–16), while B. thuringiensis can produce crystal inclusions toxic to specific insects, making it the most widely used microbial insecticide (17). For some members of the Bacillus cereus group, sporulation is not only a survival strategy to resist adversity but a prerequisite for virulence factor production. A typical example is that the insecticidal toxin from B. thuringiensis can only be produced after completion of forespore engulfment (18). The transcription of many *cry* genes, encoding insecticidal toxins such as *cry1A* (19), *cry4A* (20), *cry8E* (21), and *cry11A*, is controlled by σ^E^ and/or σ^K^ (22, 23). In addition, sporulation-specific transcription factors regulating toxins like Spo0A can positively regulate *cry1Ac* (24, 25). With progressive development of genomic data, it is found that there are differences in the functions of sporulation-related genes between the Bacillus cereus group and B. subtilis, and functional characterization of these genes may be the key to revealing differences in sporulation between these two groups (26).

Among members of the *Firmicutes*, *spoVS*, an important sporulation-related gene, is found in sporulating bacilli and clostridia but not in nonsporulating lactobacilli, listeria, staphylococci, or streptococci. In addition to *Firmicutes*, the *spoVS* gene is found in members of the bacterial phyla *Chloroflexi*, *Thermotogae*, and *Deinococcus-Thermus* (27). The distribution of the *spoVS* gene in sporulating bacteria has been investigated, and the numbers of *spoVS* homologous genes have been found to differ. There is only one gene homologous to *spoVS* in some bacteria, such as B. subtilis, Lysinibacillus sphaericus, and Clostridium difficile. Three genes homologous to *spoVS* are present in a few bacteria, such as Bacillus megaterium and Thermoanaerobacter tengcongensis. We have often found two *spoVS* homologous genes in members of the Bacillus cereus group, such as B. cereus, B. anthracis, and B. thuringiensis (Table S1 in the supplemental material). It is not clear whether these differences in the numbers of homologous genes lead to differences in the role of *spoVS* genes in the Bacillus cereus group and B. subtilis.

In this study, the two homologous *spoVS* genes were characterized in B. thuringiensis HD73. We found that different *spoVS* null mutant strains exhibited different phenotypes. In addition, the transcription of these two homologous *spoVS* genes was controlled directly by σ^H^. Functional characterization of two homologous SpoVS proteins in B. thuringiensis will help to better elucidate the formation and development of spores and provide insight into the regulation of the expression of the sporulation-dependent *cry* genes.

## RESULTS

### Identification of SpoVS1 and SpoVS2 in B. thuringiensis.

**FIG 1:**
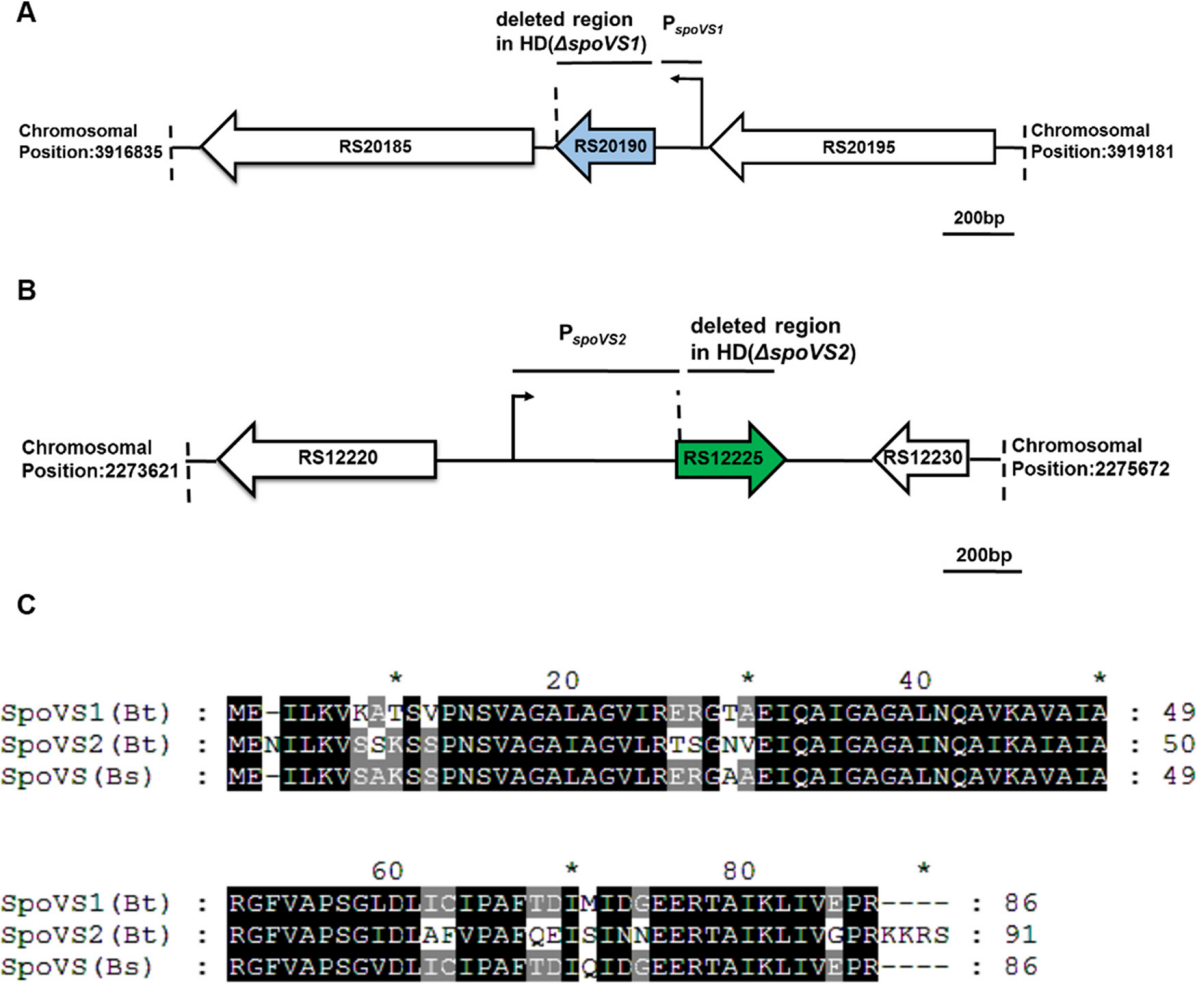
Basic description of *spoVS* genes in B. thuringiensis HD73. (A) Map of the *RS20185*–*RS20195* locus in the B. thuringiensis HD73 genome. The deleted region represents the fragment deleted in the *spoVS1* null mutant. P*_spoVS1_* is the promoter region used in the β-galactosidase activity assay. The bent arrow represents the transcription start site. ORFs are indicated by large open arrows. The scale bar corresponds to 200 bp. (B) Map of the *RS12220*–*RS12230* locus in the B. thuringiensis HD73 genome. The deleted region represents the fragment deleted in the *spoVS2* null mutant. P*_spoVS2_* is the promoter region used in the β-galactosidase activity assay. The bent arrow represents the transcription start site. ORFs are indicated by large open arrows. The scale bar corresponds to 200 bp. (C) Comparison of the amino acid sequences of three SpoVS proteins from B. thuringiensis HD73 and B. subtilis PY79. Sequence alignment of SpoVS1 in B. thuringiensis HD73 and SpoVS in B. subtilis PY79 reveals 92% identity between the two proteins. Sequence alignment of SpoVS2 in B. thuringiensis HD73 and SpoVS in B. subtilis PY79 reveals 76% identity between the two proteins. The amino acid identity between SpoVS1 and SpoVS2 is 72%.

BLASTP analysis revealed that there are two homologous *spoVS* genes in B. thuringiensis, whereas only one *spoVS* gene exists in B. subtilis. Two open reading frames (ORFs) for the SpoVS protein (HD73_RS20190 and HD73_RS12225) were identified in the B. thuringiensis HD73 genome (accession number NC_020238.1) and designated *spoVS1* and *spoVS2*. The *spoVS1* gene is located in the B. thuringiensis HD73 chromosomal genome between bp 3917898 and 3918158 and encodes a protein containing 86 amino acids (Fig. 1A). The amino acid identity between SpoVS1 in B. thuringiensis strain HD73 and SpoVS in B. subtilis strain PY79 is 92% (Fig. 1C). The *spoVS2* gene is located in the B. thuringiensis HD73 chromosomal genome between bp 2274858 and 2275133 and encodes a protein containing 91 amino acids (Fig. 1B). The amino acid identity between SpoVS2 in B. thuringiensis HD73 and SpoVS in B. subtilis PY79 is 76% (Fig. 1C). The amino acid identity between SpoVS1 and SpoVS2 is 72% (Fig. 1C).

### *spoVS1* and *spoVS2* are both σ^H^-dependent genes.

To determine the transcriptional start sites of the *spoVS1* and *spoVS2* genes, the total RNA of a wild-type B. thuringiensis HD73 strain grown in Schaeffer’s sporulation medium (SSM) to *T*_5_ (time zero [*T*_0_] is the end of the exponential growth phase, and *T_n_* is *n* hours after the end of the exponential growth phase) was extracted and 5′ rapid amplification of cDNA ends (RACE)-PCR experiment was performed. The results showed that the transcriptional start site of the *spoVS1* gene was located 46 nucleotides upstream from the *spoVS1* translational start codon (Fig. S1A). The transcriptional start site of the *spoVS2* gene was located 100 nucleotides upstream from the *spoVS2* translational start codon (Fig. S1B). To investigate whether the transcription of the *spoVS1* and *spoVS2* genes in B. thuringiensis was regulated by σ^H^, we analyzed their promoter regions in the DBTBS database (http://dbtbs.hgc.jp/). Putative σ^H^-dependent sequences of the −35 and −10 regions (RNAGGAWWW and RNNGAATWW) (21) were found in the promoter regions of the *spoVS1* (Fig. S1A) and *spoVS2* (Fig. S1B) genes.

**FIG 2:**
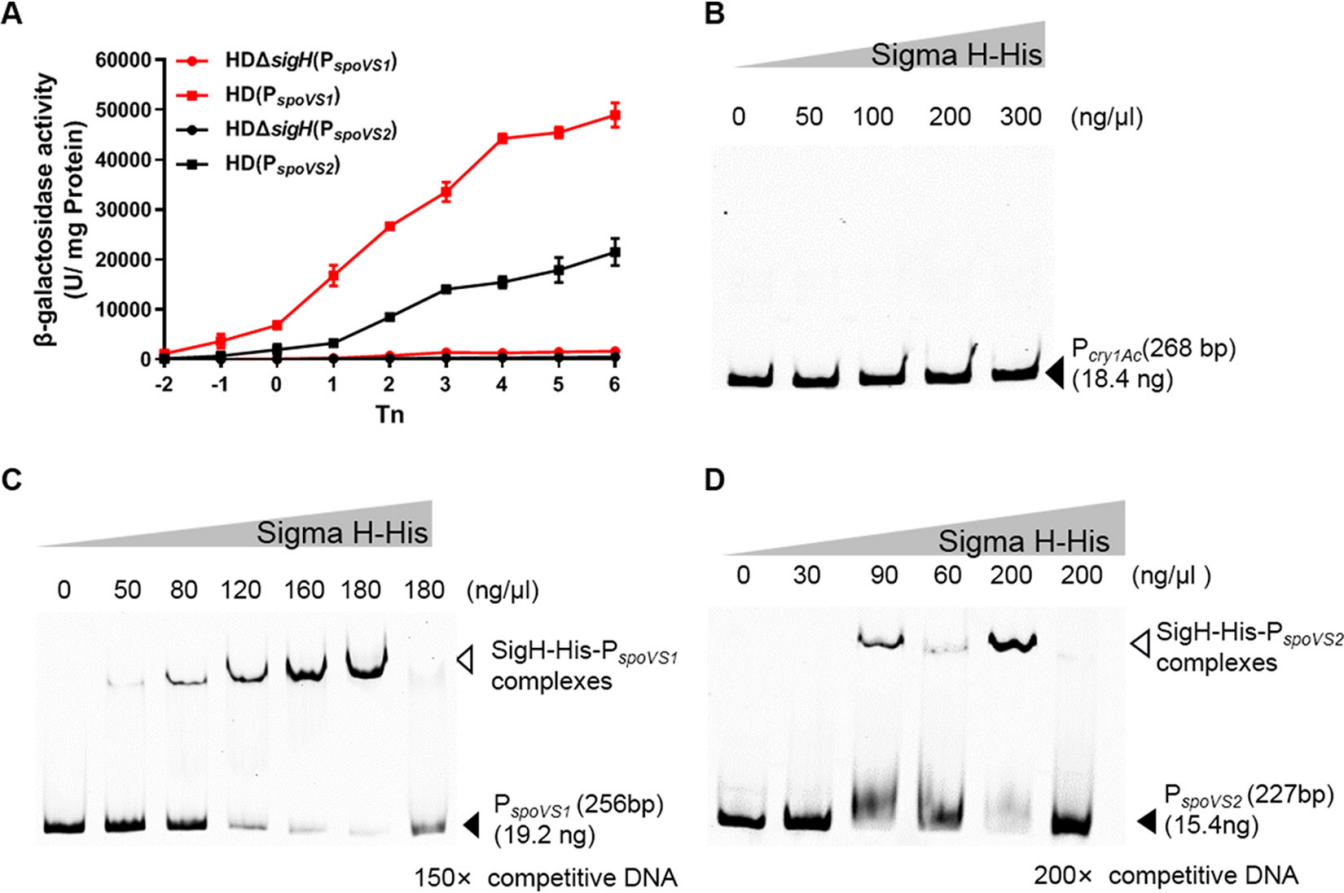
Transcriptional regulation of the *spoVS1* and *spoVS2* genes. (A) Effects of σ^H^ on P*_spoVS1_* and P*_spoVS2_* expression. β-Galactosidase activity was assessed in the wild-type B. thuringiensis HD73 (squares) and the *sigH* null mutant strains (circles) containing the plasmid-borne transcriptional fusion P*_spoVS1_*-*lacZ* (red symbols) or P*_spoVS2_*-*lacZ* (black symbols). The bacteria were grown at 30°C in SSM medium, and samples were taken at the indicated time points. Time zero (*T*_0_) is the end of the exponential growth phase, and *T_n_* is *n* hours after *T*_0_. Each data point represents the mean value from at least three independent replicates. Error bars show standard errors of the means. (B) Electrophoretic mobility shift assay (EMSA) for detecting protein-DNA interactions using FAM-labeled P*_cry1Ac_* and increasing concentrations of recombinant SigH-His. The lanes contained 0, 50, 100, 200, and 300 ng/μl of SigH-His. (C) EMSA for detecting protein-DNA interactions using FAM-labeled P*_spoVS1_* and increasing concentrations of recombinant SigH-His. The lanes contained 0, 50, 80, 120, 160, and 180 ng/μl of SigH-His. (D) EMSA for detecting protein-DNA interactions using FAM-labeled P*_spoVS2_* and increasing concentrations of recombinant SigH-His. The lanes contained 0, 30, 90, 60, and 200 ng/μl of SigH-His.

To clarify the transcriptional mechanism of the *spoVS1* and *spoVS2* genes, P*_spoVS1_* (Fig. 1A) and P*_spoVS2_* (Fig. 1B) were fused with the *lacZ* gene, and the transcriptional activities of the fusions were measured in the wild-type B. thuringiensis HD73 strain and the *sigH* null mutant HD(Δ*sigH*). The wild-type strain containing the P*_spoVS1_*-*lacZ* or P*_spoVS2_*-*lacZ* fusion displayed β-galactosidase activity from exponential phase to stationary phase and reached a maximum at *T*_6_, whereas the expression of P*_spoVS1_*-*lacZ* and P*_spoVS2_*-*lacZ* was completely blocked in the *sigH* null mutant (Fig. 2A). These results suggest that both *spoVS1* and *spoVS2* in B. thuringiensis HD73 are σ^H^-dependent genes.

To determine whether σ^H^ binds directly to the promoter regions of *spoVS1* and *spoVS2*, we expressed and purified SigH-His protein in Escherichia coli strain BL21(DE3) (Fig. S1D) and then performed an electrophoretic mobility shift assay (EMSA). First, the 268-bp FAM (6-carboxyfluorescein)-labeled fragment (FAM-P*_cry1Ac_*) (Fig. S1C) without a conserved σ^H^ motif was used as the negative control. No matter how much the concentration of purified SigH-His protein increased, the FAM-P*_cry1Ac_* fragment could not bind to it (Fig. 2B). Then, the 256-bp FAM-labeled fragment (FAM-P*_spoVS1_*) containing a conserved σ^H^ motif was incubated with increasing amounts of purified SigH-His protein. A complete shift in P*_spoVS1_* DNA fragment mobility was induced by 180 ng/μl of SigH-His protein, and the addition of 150× unlabeled specific competitor DNA restored the initial mobility of the labeled probe (Fig. 2C). A 227-bp FAM-labeled fragment (FAM-P*_spoVS2_*) containing a conserved σ^H^ motif was incubated with increasing amounts of purified SigH-His protein. An almost-complete shift in P*_spoVS2_* DNA fragment mobility was induced by 200 ng/μl of SigH-His protein, and the addition of 200× unlabeled specific competitor DNA restored the initial mobility of the labeled probe (Fig. 2D). The results of these experiments suggest that SigH protein can bind directly to the promoters of the *spoVS1* and *spoVS2* genes.

### Effects of *spoVS1* deletion on spore development and sporulation efficiency.

**FIG 3:**
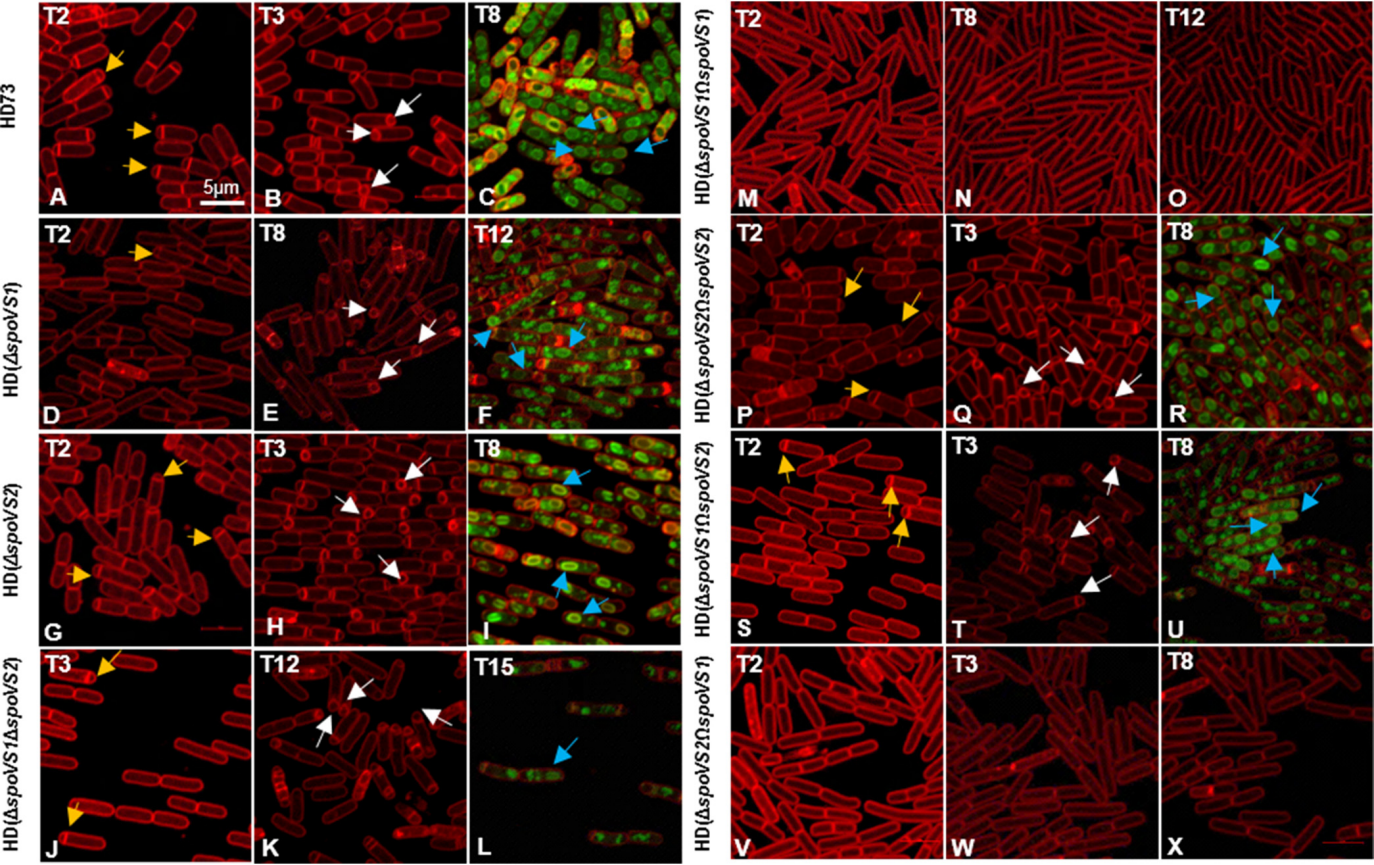
Observations of the sporulation process using a laser scanning confocal microscope. The polar septa and engulfment of B. thuringiensis HD73 (wild-type strain), HD(Δ*spoVS1*), HD(Δ*spoVS2*), HD(Δ*spoVS1*Δ*spoVS2*), HD(Δ*spoVS1*Ω*spoVS1*), HD(Δ*spoVS2*Ω*spoVS2*), HD(Δ*spoVS1*Ω*spoVS2*), and HD(Δ*spoVS2*Ω*spoVS1*) were observed using a laser scanning confocal microscope at *T*_2_, *T*_3_/*T*_8_/*T*_12_, and *T*_8_/*T*_12_/*T*_15_ after incubation in SSM at 30°C with shaking at 220 rpm. Cell membrane is visible as red fluorescence. Red lines represent membranes stained with FM 4-64, and green lines represent forespores stained with MitoTracker green FM (MTG). Yellow arrows indicate straight polar septa. White arrows indicate curved polar septa. Blue arrows indicate forespores. Scale bars = 5 μm.

Previous studies have shown that the *spoVS* mutant is delayed in the formation of polar septa but engulfs normally in B. subtilis (11). To determine the role of the *spoVS1* gene of B. thuringiensis HD73 in spore development, the *spoVS1* deletion mutant HD(Δ*spoVS1*) (Fig. 1A) was constructed by replacing the *spoVS1* coding sequence with the kanamycin (Kan) resistance gene *kan*. The deletion of *spoVS1* did not impact the growth curve of B. thuringiensis HD73 cells (Fig. S2). The polar septum formation and engulfment of wild-type B. thuringiensis HD73 and HD(Δ*spoVS1*) were observed by staining cells with the membrane-impermeable dye FM 4-64 (*N*-[3-triethylammoniumpropyl]-4-{6-[4-(diethylamino) phenyl] hexatrienyl} pyridinium dibromide) at *T*_2_ and *T*_3_ (or *T*_8_) and with membrane-permeable Mito-Tracker green at *T*_8_ (or *T*_12_) and examining them with a laser scanning confocal microscope. Cell membranes and polar septa can be dyed red using FM 4-64. Forespores can be dyed green by MitoTracker green FM dye (MTG) in the red mother cell. The results showed that polar septa were observed in many HD73 strains at *T*_2_ (Fig. 3A, yellow arrows). At this time point, only a very few cells in the HD(Δ*spoVS1*) mutants could produce the polar septum (Fig. 3D, yellow arrows). The polar septum existed in 83.4% of cells of the HD73 strain at *T*_3_ (Fig. 3B, white arrows; Table 1). During the engulfment stage, 93.5% of cells completed engulfment and formed forespores in the HD73 strain at *T*_8_ (Fig. 3C, blue arrow; Table 1). However, the polar septum formation was delayed in HD(Δ*spoVS1*). We observed polar septa in 15.1% of HD(Δ*spoVS1*) mutants at *T*_3_ (Table 1; Fig. S3A) and in 69.5% of HD(Δ*spoVS1*) mutants at *T*_8_ (Fig. 3E; Table 1), when the wild-type strain had completed engulfment (Fig. 3B), while 31.3% of the HD(Δ*spoVS1*) mutants had completed engulfment at *T*_12_ (Fig. 3F; Table 1). These results suggest that deletion of the *spoVS1* gene in B. thuringiensis delayed the formation of polar septa and caused partial cells to fail to complete engulfment.

**TABLE 1:** Effects of *spoVS1* and *spoVS2* mutations on early spore formation

Phenotype	% (no.) of sporangial cells out of total no. of cells scored in indicated strain at indicated time point^*a*^						
	HD73		HD(Δ*spoVS1*)			HD(Δ*spoVS2*)	
	*T*_3_	*T*_8_	*T*_3_	*T*_8_	*T*_12_	*T*_3_	*T*_8_
Straight and curved septa	83.4 (681)	0.9 (7)	15.1 (90)	69.5 (196)	26.4 (60)	84.1 (634)	0.4 (3)
Engulfment	2.2 (18)	93.5 (702)	0 (0)	2.1 (6)	31.3 (71)	2.4 (18)	93.6 (639)

Total no. of cells scored	817	751	594	282	227	754	683

a The percentages equal the number of cells with the indicated phenotype (sporangia with any sporulation-specific phenotype, from polar septa to engulfment) divided by the total number of cells (both vegetative cells and sporangia).

**FIG 4:**
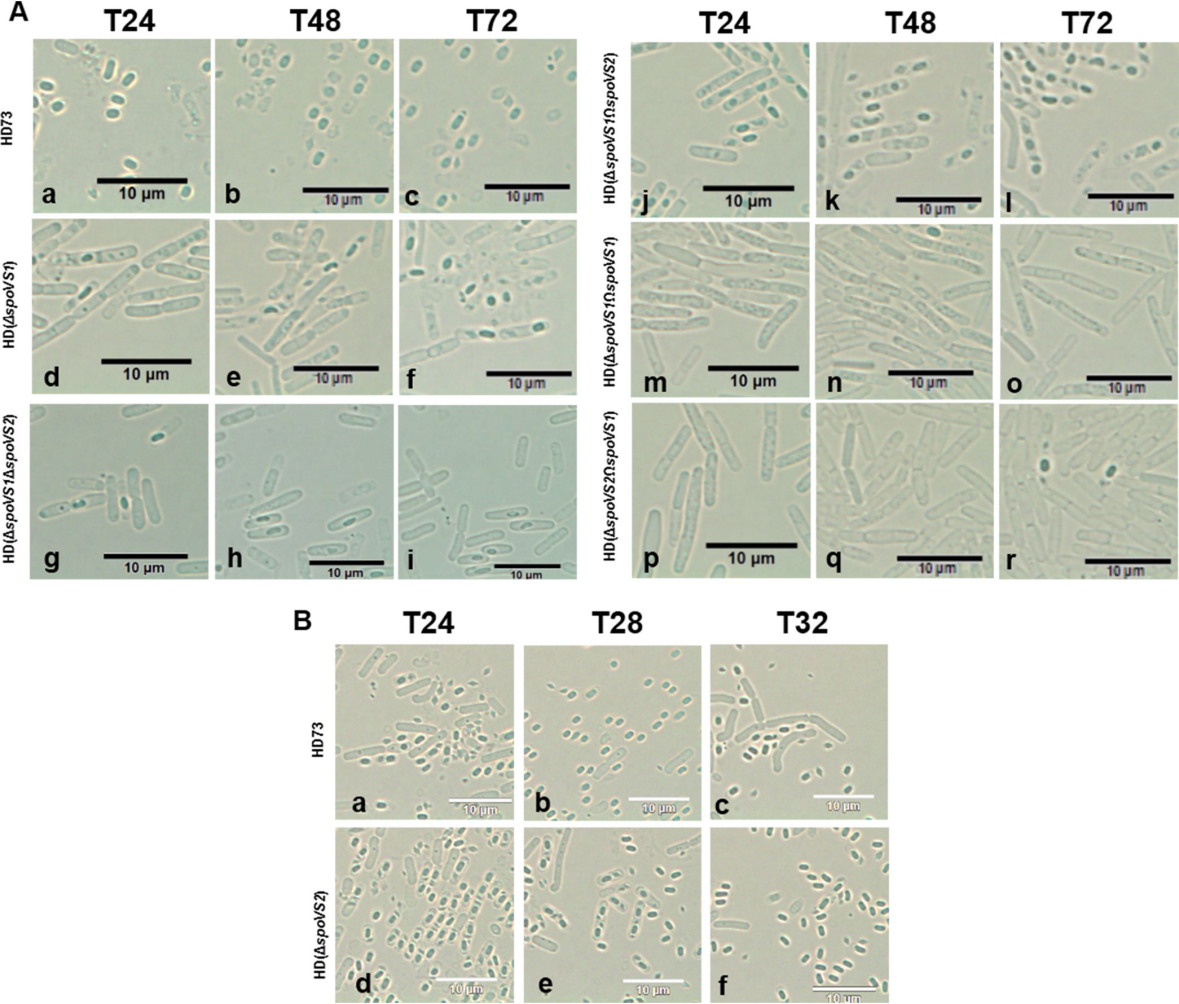
Observations of mother spore release under optical microscopy. (A) Spore release of HD73 (wild-type strain), HD(Δ*spoVS1*), HD(Δ*spoVS1*Δ*spoVS2*), HD(Δ*spoVS1*Ω*spoVS2*), HD(Δ*spoVS1*Ω*spoVS1*), and HD(Δ*spoVS2*Ω*spoVS1*). Observations were made using optical microscopy at *T*_24_, *T*_48_, and *T*_72_ after incubation in SSM at 30°C with shaking at 220 rpm. Scale bars = 10 μm. (B) Spore release of HD73 (wild-type strain) and HD(Δ*spoVS2*) mutant. Observations were made using optical microscopy at *T*_24_, *T*_28_, and *T*_32_ after incubation in SSM at 30°C with shaking at 220 rpm. Scale bars = 10 μm.

**FIG 5:**
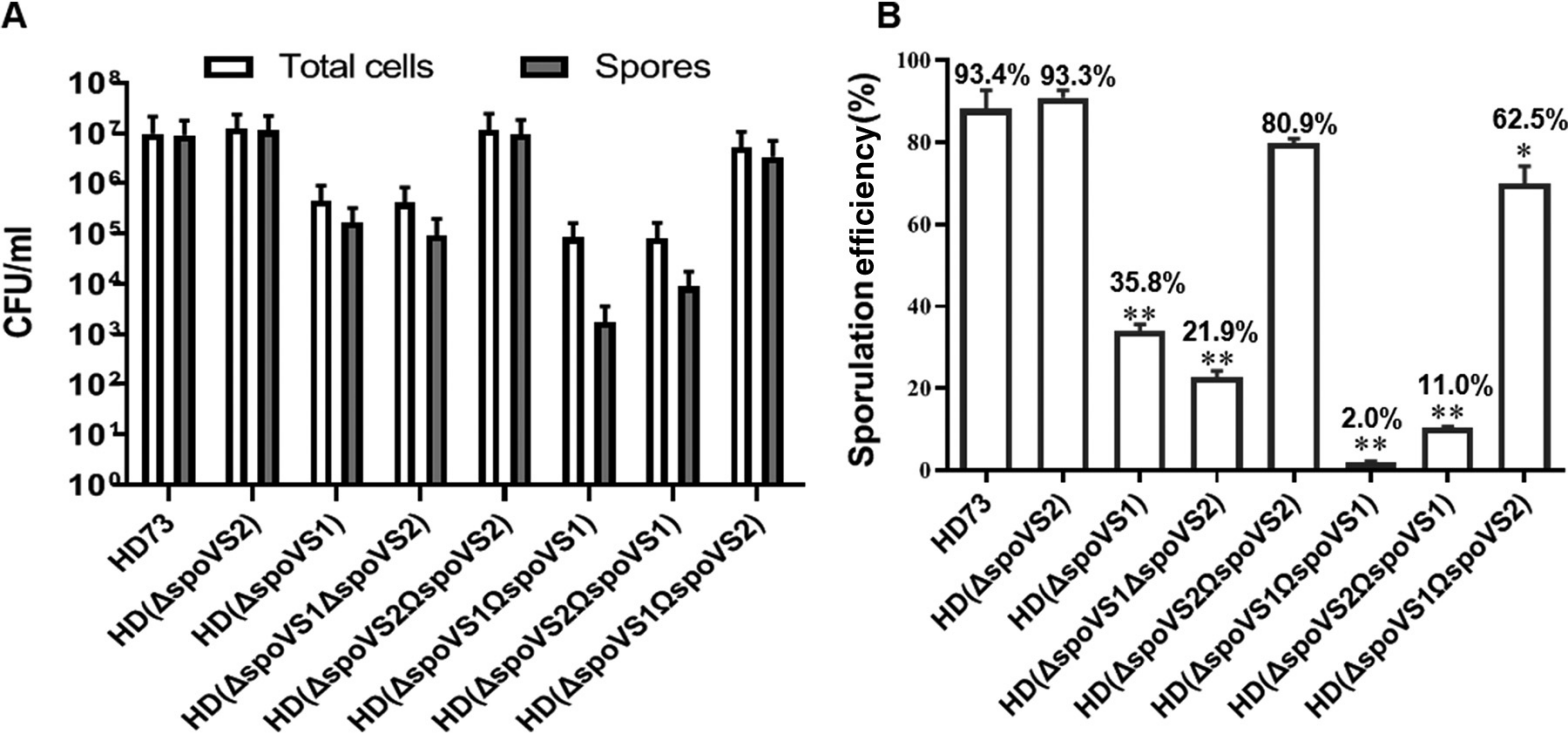
Comparison of the sporulation frequencies of HD73 (wild-type strain), HD(Δ*spoVS2*), HD(Δ*spoVS1*), HD(Δ*spoVS1*Δ*spoVS2*), HD(Δ*spoVS2*Ω*spoVS2*), HD(Δ*spoVS1*Ω*spoVS1*), HD(Δ*spoVS2*Ω*spoVS1*), and HD(Δ*spoVS1*Ω*spoVS2*). (A) The counts of total cells and spores in HD73, HD(Δ*spoVS1*), HD(Δ*spoVS2*), HD(Δ*spoVS1*Δ*spoVS2*), HD(Δ*spoVS2*Ω*spoVS2*), HD(Δ*spoVS1*Ω*spoVS1*), HD(Δ*spoVS2*Ω*spoVS1*), and HD(Δ*spoVS1*Ω*spoVS2*). Deletion of the *spoVS1* gene reduced the numbers of cells and spores, but deletion of the *spoVS2* gene did not impact the numbers of cells and spores. Self-complement of *spoVS1* reduced the numbers of cells and spores, but the complement of the *spoVS2* gene to the HD(Δ*spoVS1*) mutant could restore the total numbers of cells and spores. Each bar represents the mean value from at least three independent replicates. The error bars represent standard deviations. (B) The sporulation frequencies of all strains whose total cell counts and spore counts are shown in panel A. The sporulation frequency was defined as the ratio of the number of spores to the total number of cells, multiplied by 100. The percentage represents the average sporulation frequency. The sporulation frequency of every *spoVS*-related strain was compared with that of the wild-type strain (HD73), and the data were analyzed with SPSS (version 19.0) using the *t* test (*, *P* ≤ 0.05; **, *P* ≤ 0.01). The error bars represent standard deviations.

We used optical microscopy to observe the long-term culture. At *T*_24_, almost all of the spores were mature and had been released in wild-type B. thuringiensis HD73 (Fig. 4Aa); however, the release of spores was infrequently observed in the HD(Δ*spoVS1*) mutant at *T*_24_, *T*_48_, and *T*_72_ (Fig. 4Ad, e, and f). These results suggest that deletion of the *spoVS1* gene in B. thuringiensis blocked spore release. In addition, consistent with the failure of some cells to complete engulfment, we observed that not all cells of HD(Δ*spoVS1*) were able to produce spores (Fig. 4Ad, e, and f). To determine the effect of deletion of *spoVS1* on sporulation efficiency, we performed spore count experiments on the wild-type B. thuringiensis HD73 and the mutant strain HD(Δ*spoVS1*) at *T*_24_. We found that the total cell counts and spore counts for mutant strains HD(Δ*spoVS1*) were approximately 2 orders of magnitude lower than those of the wild-type strain [Fig. 5A, HD73 and HD(ΔspoVS1)]. The sporulation frequency of the wild type was 93.4% (Fig. 5B, HD73). The sporulation frequency of HD(Δ*spoVS1*) was a third of that of the wild type, 35.8% [Fig. 5B, HD(Δ*spoVS1*)]. These results suggest that deletion of the *spoVS1* gene reduced total cell numbers, spore numbers, and sporulation frequency.

### Effects of *spoVS*2 deletion on spore development and sporulation efficiency.

To determine the role of the *spoVS2* genes of B. thuringiensis HD73 in spore development, the *spoVS2* deletion mutant HD(Δ*spoVS2*) (Fig. 1A) was constructed by replacing the *spoVS2* coding sequence with the kanamycin (Kan) resistance gene *kan*. The deletion of *spoVS2* did not impact the growth curve of B. thuringiensis HD73 cells (Fig. S2). Laser scanning confocal microscopy observation showed that the proportions of polar septa (*T*_3_) and forespores (*T*_8_) were 84.1% and 93.6%, respectively, in the HD(Δ*spoVS2*) strain. (Fig. 3H, white arrows, and I, blue arrows; Table 1). This was not different from the results for the wild type (Fig. 3B, white arrows and C, blue arrows; Table 1). These results suggest that deletion of the *spoVS2* gene in B. thuringiensis did not affect the formation of polar septa.

Optical microscopy observation showed that at *T*_24_, only a very small number of spores were released in HD(Δ*spoVS2*) (Fig. 4Bd), while the spores were almost completely released in the wild-type strain (Fig. 4Ba). Half of the spores of the HD(Δ*spoVS2*) strain were not released at *T*_28_ (Fig. 4Be), while all of the spores were released in HD(Δ*spoVS2*) at *T*_32_ (Fig. 4Bf). These results suggest that deletion of the *spoVS2* gene in B. thuringiensis delayed spore release. The spore count results showed that the total cell and spore counts of HD(Δ*spoVS2*) were not different from those of the wild type [Fig. 5A, HD73 and HD(*ΔspoVS2*)]. The sporulation frequency of the HD(Δ*spoVS2*) mutant (93.3%) [Fig. 5B, HD(Δ*spoVS2*)] was also consistent with that of the wild type (93.4%) (Fig. 5B, HD73). These results suggest that deletion of the *spoVS2* gene did not affect total cell numbers, spore numbers, or sporulation frequency. However, deletion of the *spoVS2* gene in B. thuringiensis delayed the spore release.

### The functions of SpoVS1 and SpoVS2 were partially redundant.

Prior to this analysis, we had a preliminary understanding of the functions of the *spoVS1* and *spoVS2* genes in B. thuringiensis and observed similarities and differences with *spoVS* in B. subtilis. To clarify the functional relationship of *spoVS1* and *spoVS2* in B. thuringiensis, the double mutant strain HD(Δ*spoVS1*Δ*spoVS2*) was constructed by deleting the *spoVS2* coding sequence in the HD(Δ*spoVS1*) mutant. We introduced pHT*spoVS1* and pHT*spoVS2* vectors into the HD(Δ*spoVS1*) and HD(Δ*spoVS2*) mutants to obtain the recombinant strains HD(Δ*spoVS1*Ω*spoVS1*) and HD(Δ*spoVS2*Ω*spoVS2*). We further introduced pHT*spoVS1* and pHT*spoVS2* vectors into the HD(Δ*spoVS2*) and HD(Δ*spoVS1*) mutants to obtain the recombinant strains HD(Δ*spoVS1*Ω*spoVS2*) and HD(Δ*spoVS2*Ω*spoVS1*). The growth curves of the double mutant strain and the recombinant strains were similar to the growth curve of wild-type B. thuringiensis HD73 (Fig. S2).

Laser scanning confocal microscopy showed that polar septa were present in 4.0% of the double mutant strain at *T*_3_ [at which time they were observed in 15.1% of HD(Δ*spoVS1*) cells] (Fig. 3J; Table 2). Engulfment was observed in 25.2% of the double mutant strain at *T*_15_ (Fig. 3L; Table 2). The engulfment of the double mutant (Fig. 3L) was later than that of the wild type, HD(Δ*spoVS2*), or HD(Δ*spoVS1*) (Fig. 3C, F, and I). Otherwise, polar septa were observed in 51.9% of HD(Δ*spoVS1*Ω*spoVS2*) at *T*_3_ (Fig. 3T; Table 2), and engulfment was observed in 48.6% of HD(Δ*spoVS1*Ω*spoVS2*) at *T*_8_ (Fig. 3U; Table 2). We found that the delay in polar septum formation was more apparent in the double mutant strain HD(Δ*spoVS1*Δ*spoVS2*) than in the single mutant strain HD(Δ*spoVS1*). The effect of *spoVS1* deletion on polar septum delay may be compensated by *spoVS2*.

**TABLE 2:** Effects of HD(Δ*spoVS1*Δ*spoVS2*) and HD(Δ*spoVS1*Ω*spoVS2*) on early spore formation

Phenotype	% (no.) of sporangial cells out of total no. of cells scored in indicated strain at indicated time point^*a*^			
	HD(Δ*spoVS1*Δ*spoVS2*)		HD(Δ*spoVS1*Ω*spoVS2*)	
	*T*_3_	*T*_15_	*T*_3_	*T*_8_
Septa	4.0 (9)		51.9 (191)	
Engulfment		25.2 (58)		48.6 (120)

Total no. of cells scored	277	230	368	247

a The percentages equal the number of cells with the indicated phenotype (sporangia with any sporulation-specific phenotype, from polar septa to engulfment) divided by the total number of cells (both vegetative cells and sporangia).

Optical microscopy observation showed that the total cell and spore counts for double mutant HD(Δ*spoVS1*Δ*spoVS2*) were approximately 2 orders of magnitude lower than those of the wild-type strain [Fig. 5A, HD(Δ*spoVS1*Δ*spoVS2*)]. Only 21.9% spores were observed in the double mutant HD(Δ*spoVS1*Δ*spoVS2*) at *T*_24_, less than in the HD(Δ*spoVS1*) mutant (35.8%) [Fig. 5B, HD(Δ*spoVS1*Δ*spoVS2*) and HD(Δ*spoVS1*)], while 62.5% spores were observed in the HD(Δ*spoVS1*Ω*spoVS2*) strain at *T*_24_ [Fig. 5B, HD(Δ*spoVS1*Δ*spoVS2*)]. We found that the decrease of sporulation efficiency was more apparent in the double mutant strain HD(Δ*spoVS1*Δ*spoVS2*) than in the single mutant strain HD(Δ*spoVS1*). However, the sporulation frequency resulting from the absence of *spoVS1* could be partially compensated by *spoVS2* but could not be restored to the level of the wild type. Further evidence indicated that the functions of the two homologous *spoVS* genes were partially redundant in B. thuringiensis.

### Mutation of *spoVS1* or *spoVS2* has an effect on the transcription activities of pro-*sigE* and *spoIIE*.

**FIG 6:**
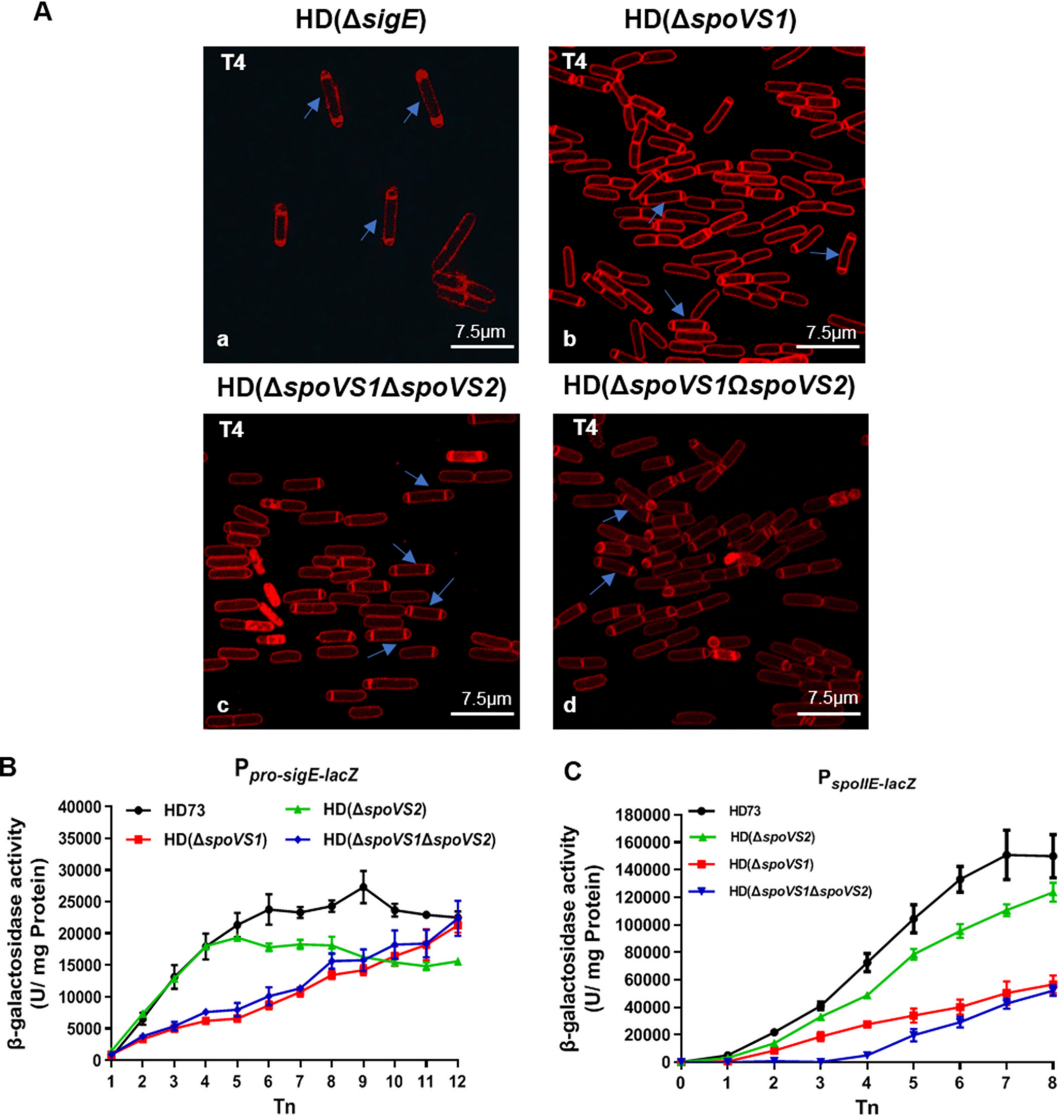
Effects of various mutations on pro-*sigE* and *spoIIE* expression. (A) Disporic septa of HD(Δ*sigE*) mutant (a), HD(Δ*spoVS1*) mutant (b), HD(Δ*spoVS1*Δ*spoVS2*) double mutant (c), and HD(Δ*spoVS1*Ω*spoVS2*) mutant (d) were observed using a laser scanning confocal microscope at *T*_4_ after incubation in SSM at 30°C with shaking at 220 rpm. Cell membranes are visible as red fluorescence. Red lines represent membranes stained with FM 4-64. Blue arrows indicate disporic septa. Scale bars = 7.5 μm. (B) β-Galactosidase activities were assessed for HD73 (wild-type) (black circles), HD(Δ*spoVS1*) mutant (red squares), HD(Δ*spoVS2*) mutant (green triangles), and HD(Δ*spoVS1*Δ*spoVS2*) mutant (blue rhombuses) containing the plasmid-borne transcriptional fusion P*_pro_*_-_*_sigE_*-*lacZ*. The bacteria were grown at 30°C in SSM medium, and samples were taken at the indicated time points. *T*_0_ is the end of the exponential growth phase, and *T_n_* is *n* hours after *T*_0_. Each data point represents the mean value from at least three independent replicates. Error bars show the standard errors of the means. (C) β-Galactosidase activity was assessed for HD73 (wild-type) (black circles), HD(Δ*spoVS1*) mutant (red squares), HD(Δ*spoVS2*) mutant (green triangles), and HD(Δ*spoVS1*Δ*spoVS2*) mutant (blue rhombuses) containing the plasmid-borne transcriptional fusion P*_spoIIE_*-*lacZ*. The bacteria were grown at 30°C in SSM medium, and samples were taken at the indicated time points. *T*_0_ is the end of the exponential growth phase, and *T_n_* is *n* hours after *T*_0_. Each data point represents the mean value from at least three independent replicates. Error bars show the standard errors of the means.

During laser scanning confocal microscopy observations, we found that some cells produced 13.9% disporic septa in the mutant strain HD(Δ*spoVS1*), 12.4% disporic septa in HD(Δ*spoVS1*Δ*spoVS2*), and 7.1% disporic septa in the complemented strain HD(Δ*spoVS1*Ω*spoVS2*) at *T*_4_ (Fig. 6Ab, c, and d; Table 3). Disporic septa are a typical feature of *sigE* mutants of B. subtilis (28). In addition, this phenomenon occurs when the regulons of σ^E^, *spoIID*, *spoIIM*, and *spoIIP* are absent simultaneously (28). The *sigE* mutant of B. thuringiensis also had a disporic-septum phenotype (Fig. 6Aa). This drew our attention to the question of whether σ^E^ could work properly in a series of mutants of *spoVS*. In order to investigate whether this particular phenotype was caused by the decreased expression of pro-*sigE* in these strains, a plasmid containing the P_pro-_*_sigE_*-*lacZ* fusion was introduced into the wild-type strain and the mutant strains HD(Δ*spoVS1*), HD(Δ*spoVS2*), and HD(Δ*spoVS1*Δ*spoVS2*). β-Galactosidase activity assays showed that the expression of pro-*sigE* in the wild-type strain started at *T*_1_, increased from *T*_1_ to *T*_6_, and then trended to stabilization (Fig. 6B, black circles). The expression of pro-*sigE* in the mutant strain HD(Δ*spoVS2*) was similar to that in the wild-type strain before *T*_4_, while it was lower than in the wild-type strain from *T*_4_ (Fig. 6B, green triangles). The expression of pro-*sigE* in the mutant strains HD(Δ*spoVS1*) and HD(Δ*spoVS1*Δ*spoVS2*) increased slowly from *T*_1_, and the transcription activity of pro-*sigE* was almost identical to that of the wild-type strain at *T*_12_ (Fig. 6, red squares and blue rhombuses). Our results confirm that the expression of pro-*sigE* decreased differently in the HD(Δ*spoVS1*) and HD(Δ*spoVS2*) strains. The expression of pro-*sigE* decreased from the initial stage of sporulation in HD(Δ*spoVS1*) and HD(Δ*spoVS1*Δ*spoVS2*). However, the decrease in the expression of pro-*sigE* in HD(Δ*spoVS2*) only manifested after the *T*_4_ stage of forespore engulfment.

**TABLE 3:** Effects of *spoVS1* mutations on polar septa

Phenotype	% (no.) of cells with polar septa out of total no. of cells in indicated strain at *T*_4_^*a*^		
	HD(Δ*spoVS1*)	HD(Δ*spoVS1*Δ*spoVS2*)	HD(Δ*spoVS1*Ω*spoVS2*)
Straight and curved septa	26.3 (85)	19.7 (62)	51.3 (225)
Disporic septa	13.9 (45)	12.4 (39)	7.1 (31)

Total no. of cells scored	323	314	439

a The percentages equal the number of cells with polar septa of the indicated phenotype (straight and curved septa or disporic) divided by the total number of cells (both vegetative cells and sporangia).

In addition, it has been reported that the disporic-septum phenomenon was also observed in the Δ*spoIIE* mutant of B. subtilis (29). Therefore, we also detected *spoIIE* expression in the wild-type strain and the mutant strains HD(Δ*spoVS1*), HD(Δ*spoVS2*), and HD(Δ*spoVS1*Δ*spoVS2*). β-Galactosidase activity assays showed that the expression of *spoIIE* in the wild-type strain started at *T*_0_, increased from *T*_1_ to *T*_7_, and then trended to stabilization (Fig. 6C, black circles), and the expression levels of *spoIIE* in the mutant strains HD(Δ*spoVS1*), HD(Δ*spoVS2*), and HD(Δ*spoVS1*Δ*spoVS2*) were lower than in the wild-type strain from *T*_1_ to *T*_8_ (Fig. 6C). The decrease of *spoIIE* expression was more significant in HD(Δ*spoVS1*) than in HD(Δ*spoVS2*) (Fig. 6C, red squares and green triangles). The expression of *spoIIE* in the HD(Δ*spoVS1*Δ*spoVS2*) mutant started at *T*_3_, and the transcription activity of *spoIIE* was almost identical to that of HD(Δ*spoVS1*) at *T*_8_ (Fig. 6C, blue rhombuses). Our results confirm that the expression of *spoIIE* decreased similarly in HD(Δ*spoVS1*) and HD(Δ*spoVS2*), but the decrease was more significant in the HD(Δ*spoVS1*). The initiation of *spoIIE* expression in the double mutant was delayed by 3 h compared with that in the two single mutants and the wild type, which should be due to the superimposed effects of *spoVS1* and *spoVS2* deletion. These results suggest that the disporic-septum phenotype is caused by significant inhibition of pro-*sigE* and *spoIIE* transcription in the HD(Δ*spoVS1*) and HD(Δ*spoVS1*Δ*spoVS2*) mutants.

## DISCUSSION

We report the identification and characterization of two genes homologous to *spoVS* of B. subtilis in B. thuringiensis, *spoVS1* and *spoVS2*. As in B. subtilis, both *spoVS1* and *spoVS2* in B. thuringiensis are controlled by the σ^H^ factor. In B. subtilis, the *spoVS* mutant is delayed in the formation of polar septa and is blocked in stage V of sporulation with a spore coat defect (10, 11). In B. thuringiensis, the deletion of the *spoVS1* gene delays the formation of polar septa, reduces the numbers of total cells and spores, decreases the sporulation efficiency, and blocks spore release, while the effect of deletion of the *spoVS2* gene on the spore development process is mainly reflected in the delay of spore release. The deletion of the *spoVS1* gene produces a spore development phenotype similar to that of the *spoVS* mutant of B. subtilis. Different from the deletion of *spoVS* in B. subtilis, the deletion of the *spoVS1* gene can cause the occurrence of disporic septa in a certain proportion of cells (Fig. 6Ab, c, and d; Table 3) and failure to complete engulfment in some cells (Table 1). Moreover, the delay in polar septum formation is more apparent in the double mutant strain HD(Δ*spoVS1*Δ*spoVS2*) than in the single mutant strain HD(Δ*spoVS1*). The effect of *spoVS1* deletion on polar septum delay may be compensated by *spoVS2* overexpression. The results suggest that the functions of the two homologous *spoVS* genes have a partial overlap in polar septum development and spore maturation. In addition, we fortuitously found that it was almost impossible to observe spore production in HD(Δ*spoVS1*Ω*spoVS1*) and HD(Δ*spoVS2*Ω*spoVS1*). It is possible that both high (overexpression) and low (deletion) levels of SpoVS1 expression can affect the expression of key genes in the sporulation network.

The formation of the polar septum is the first significant change of cell morphology during spore formation. The mutations of stage II loci block morphological development from proceeding beyond the polar septum step (29). As suggested by the phenotypic analysis, we also examined the expression of several key sporulation genes in *spoVS* series mutants. σ^E^ regulates at least 253 genes with different functions in B. subtilis (7). These include pro-*sigK* and some genes related to forespore engulfment, such as *spoIID*, *spoIIM*, and *spoIIP*, causing cell growth to be blocked to stage II of sporulation when they are absent (7–9). Disporic septa are a typical feature of *sigE* mutants. Disporic septa also occur when *spoIID*, *spoIIM*, and *spoIIP* are absent simultaneously (28). In addition, σ^E^ regulates some proteins related to the synthesis of cortex and coat, such as *spoVB*, *spoVD*, and *spoVE* (30–32). The deletion of these genes affects the maturation and release of spores. SpoIIE is also involved in polar septum formation in B. subtilis (5). Mutations of *spoIIE* block sporulation at the stage of polar septum formation and prevent the activation of σ^F^. In addition, *spoIIE* mutants produce different polar septum phenotypes, such as no septa, thick septa, and disporic septa (29). Our results show that the transcription levels of pro-*sigE* and *spoIIE* are greatly inhibited in the HD(Δ*spoVS1*) mutant. This provides a reasonable explanation for the phenotype caused by the deletion of *spoVS1*. However, the transcription level of *spoIIE* also decreases in the HD(Δ*spoVS2*) mutant, but the degree of decrease does not affect the role of *spoIIE* in polar septum formation. The expression of pro-*sigE* in the HD(Δ*spoVS2*) mutant is not affected before *T*_4_, but it is inhibited after *T*_4_. Combined with the phenotype experiments, the results show that the deletion of *spoVS2* does not significantly inhibit genes associated with polar septum and forespore formation, but the cumulative effects result in a longer time for maturation of the spore.

The production of Cry1Ac in *spoVS1* and *spoVS2* mutants is consistent with the effects of their mutations on sporulation. In B. thuringiensis, σ^E^ and σ^K^ directly determine the transcription of the *cry1Ac* gene under the guidance of the BtI and BtII promoters, respectively (33). Our results show that Cry1Ac production is reduced to different degrees in the HD(Δ*spoVS1*) and HD(Δ*spoVS2*) mutants (Fig. S3). In HD(Δ*spoVS1*), the transcription activities of pro-*sigE* (Fig. 6B, red squares) and pro-*sigK* (Fig. S4, red squares) are significantly reduced. Therefore, the very low Cry1Ac production may be caused by the decrease of σ^E^ and σ^K^ in HD(Δ*spoVS1*). However, the lower level of Cry1Ac production than in the wild-type strain may be related to the decrease of σ^E^ in HD(Δ*spoVS2*). In HD(Δ*spoVS2*), the transcription activity of pro-*sigE* was reduced after *T*_4_ (Fig. 6B, green triangles), while the transcription activity of pro-*sigK* was 1 h earlier than in the wild-type strain (Fig. S4, green triangles). It has been confirmed that production of σ^K^ about 1 h earlier than normal does negatively regulate *sigE* expression in B. subtilis (34).

We found that these two homologous *spoVS* genes were highly conserved in the Bacillus cereus group. The amino acid sequence of SpoVS1 shared 100% identity with the homologous genes in the Bacillus cereus group, and the SpoVS2 showed 78% to 100% sequence identities with its homologous genes (Table S2). Therefore, our findings for *spoVS1* and *spoVS2* genes in B. thuringiensis can be extended to the entire Bacillus cereus group.

## MATERIALS AND METHODS

### Bacterial strains, plasmids, and growth conditions.

The bacterial strains and plasmids used in this study are listed in Table 4. E. coli strains were cultured at 37°C in Luria-Bertani (LB) medium (35). B. thuringiensis strains were cultured at 30°C in Schaeffer’s sporulation medium (SSM) (36). Time zero was defined as the beginning of the transition phase between the exponential and stationary phases. The antibiotic concentrations used for bacterial selection were as follows: 100 μg/ml ampicillin for E. coli and 5 μg/ml erythromycin or 100 μg/ml kanamycin for B. thuringiensis.

**TABLE 4:** Strains and plasmids used in this study

Strain or plasmid		Characteristic(s)	Source
E. coli strains			
TG1		Δ(*lac-proAB*) *supE thi hsd-5* (F′ *traD36 proA^+^ proB^+^ lacI*^q^ *lacZ*ΔM15)	40
ET		*F*^−^ *dam-13*::Tn*9 dcm-6 hsdM-hsdR recF143 zjj-202*::Tn*10 galK2 galT22 ara14 pacY1 xyl-5 leuB6 thi-1*	40
BL21(DE3)		*F^−^ dcm ompT hsdS* (*r_B_*^−^ *m_B_*^−^) *galλ*(DE3)	48
BL21(pET*sigH*)		BL21(DE3) with pETsigH plasmid	This study

B. thuringiensis strains			
HD73		Wild type containing *cry1Ac* gene	
HD(Δ*sigH*)		HD73 Δ*sigH* mutant	21
HD(P*spoVS1*-*lacZ*)		HD73 strain containing plasmid P*spoVS1*-*lacZ*	This study
HD(P*spoVS2*-*lacZ*)		HD73 strain containing plasmid P*spoVS2*-*lacZ*	This study
HDΔ*sigH*(P*spoVS1*-*lacZ*)		Δ*sigH* mutant containing plasmid P*spoVS1*-*lacZ*	This study
HDΔ*sigH*(P*spoVS2*-*lacZ*)		Δ*sigH* mutant containing plasmid P*spoVS2*-*lacZ*	This study
HD(Δ*spoVS1*)		HD73 Δ*spoVS1* mutant	This study
HD(Δ*spoVS2*)		HD73 Δ*spoVS*2 mutant	This study
HD(Δ*spoVS1*Δ*spoVS2*)		HD73 Δ*spoVS1* Δ*spoVS2* double mutant	This study
HD(Δ*spoVS1*Ω*spoVS1*)		HD73 Δ*spoVS1* mutant containing plasmid pHTHF*spoVS1*	This study
HD(Δ*spoVS2*Ω*spoVS2*)		HD73 Δ*spoVS2* mutant containing plasmid pHTHF*spoVS2*	This study
HD(Δ*spoVS1*Ω*spoVS2*)		HD73 Δ*spoVS1* mutant containing plasmid pHTHF*spoVS2*	This study
HD(Δ*spoVS2*Ω*spoVS1*)		HD73 Δ*spoVS2* mutant containing plasmid pHTHF*spoVS1*	This study
HD(Δ*sigE*)		HD73 Δ*sigE* mutant	24
HD(P*sigE*-*lacZ*)		HD73 strain containing plasmid P*sigE*-*lacZ*	44
HDΔ*spoVS1*(P*sigE-lacZ*)		HD73 Δ*spoVS1* mutant containing plasmid P*sigE-lacZ*	This study
HDΔ*spoVS2*(P*sigE*-*lacZ*)		HD73 Δ*spoVS2* mutant containing plasmid P*sigE-lacZ*	This study
HDΔ*spoVS1*Δ*spoVS2*(P*sigE*-*lacZ*)		HD73 Δ*spoVS1* Δ*spoVS2* mutant containing plasmid P*sigE-lacZ*	This study
HD(P*sigK*-*lacZ*)		HD73 strain containing plasmid P*sigK*-*lacZ*	44
HDΔ*spoVS1*(P*sigK-lacZ*)		HD73 Δ*spoVS1* mutant containing plasmid P*sigK-lacZ*	This study
HDΔ*spoVS2*(P*sigK*-*lacZ*)		HD73 Δ*spoVS2* mutant containing plasmid P*sigK-lacZ*	This study
HDΔ*spoVS1*Δ*spoVS2*(P*sigK*-*lacZ*)		HD73 Δ*spoVS1* Δ*spoVS2* mutant containing plasmid P*sigK-lacZ*	This study

Plasmids			
pHT304-18Z		Promoterless *lacZ* vector, Ery^r^ Amp^r^; 9.7 kb	39
pET-21b		Expression vector, Amp^r^; 5.4 kb	Novagen
pMAD		Amp^r^ Ery^r^, temp-sensitive B. thuringiensis*-*E. coli shuttle vector	41
P*spoVS1*-*lacZ*		pHT304-18Z carrying P*spoVS1*, Amp^r^ Ery^r^	This study
P*spoVS2*-*lacZ*		pHT304-18Z carrying P*spoVS2*, Amp^r^ Ery^r^	This study
P*sigE*-*lacZ*		pHT304-18Z carrying P*sigE*, Amp^r^ Ery^r^	44
P*sigK*-*lacZ*		pHT304-18Z carrying P*sigK*, Amp^r^ Ery^r^	44
pET*sigH*		pET-21b containing *sigH* gene, Amp^r^	This study
pMADΩ*spoVS1*::*Km*		pMAD carrying partial *spoVS1* deletion gene Ω *Km* gene	This study
pMADΩ*spoVS2*:: *Km*		pMAD carrying partial *spoVS2* deletion gene Ω *Km* gene	This study
pMADΩ*spoVS2*		pMAD carrying partial *spoVS2* deletion gene	This study
pHT315		B. thuringiensis*-*E. coli shuttle vector	43
pHT*spoVS1*		pHT315 carrying *spoVS1*, Amp^r^ Ery^r^	This study
pHT*spoVS2*		pHT315 carrying *spoVS2*, Amp^r^ Ery^r^	This study

### DNA manipulation and transformation.

Reagents and methods for PCR amplification and purification have been described previously (21). Chromosomal DNA was extracted from B. thuringiensis as described previously (37). Restriction enzymes and T4 DNA ligase (TaKaRa Biotechnology Corporation, Dalian, China) were employed according to the manufacturer’s instructions. Oligonucleotide primers (Table 5) were synthesized by Sangon (Beijing, China). Plasmid DNA was extracted from E. coli using a plasmid extraction kit (Axgen Biotechnology Corporation, Hangzhou, China). After agarose gel electrophoresis, all DNA fragments were isolated and purified using an AxyPrep DNA gel extraction kit (Axygen). All constructs were confirmed by PCR followed by DNA sequencing (BGI, Beijing, China). Standard procedures were used for E. coli transformation. B. thuringiensis cells were transformed using electroporation as previously described (38).

**TABLE 5:** Primers and sequences used in this study

Primer	Sequence (5′–3′)
spoVS1RACE-R	CGCTGGGATACAAATCAGGTCCAAACCACTAGGCGC
spoVS2RACE-R	CCACTTGGAGCGACGAACCCTCTTGCAATCGC
spoVS1-a	AGATCTATCGATGCATGCCATGGTACCCGGGAGCTTATCTTTGTGAACTGTAATGG
spoVS1-b	CCTATCACCTCAAATGGTTCGCTGGTAGAACCTCGTTAAGTTTAAACAAC
S1Km-a	GTTGTTTAAACTTAACGAGGTTCTACCAGCGAACCATTTGAGGTGATAGG
S1Km-b	CTGCAAACAGGTTGTTTAAACTTAAAATTCCTCGTAGGCGCTCG
spoVS1-c	CGAGCGCCTACGAGGAATTTTAAGTTTAAACAACCTGTTTGCAG
spoVS1-d	GCGTCTGCAGAAGCTTCTAGAATTCGAGCTCCACGTGGGTGCTCGCAAAGTTTCA
spoVS2-a	AGATCTATCGATGCATGCCATGGTACCCGGGAGTTTGAAGAATGATAGTATGAACCCAAA
spoVS2-b	CCTCAAATGGTTCGCTGTTCCATGTAAGTTGCTCCCTCTATT
S2Km-a	AATAGAGGGAGCAACTTACATGGAACAGCGAACCATTTGAGG
S2Km-b	CCCTTATAGTAACGCTCTCTTCTACTAAAATTCCTCGTAGGCGCTCG
spoVS2-c	CGAGCGCCTACGAGGAATTTTAGTAGAAGAGAGCGTTACTATAAGGG
spoVS2-d	GCGTCTGCAGAAGCTTCTAGAATTCGAGCTCCAAGAATAAAGCTCTCATTCAACGC
spoVS1-F	GCATGCCTGCAGGTCGACTCTAGAGCCATGCGGAGACTACAAGTGA
spoVS1-R	TGTAAAACGACGGCCAGTGAATTCGTTGTTTAAACTTAACGAGGTTCTAC
spoVS2-F	GCATGCCTGCAGGTCGACTCTAGAGCGAGTTTGAAGAATGATAGTATGAACCC
spoVS2-R	TGTAAAACGACGGCCAGTGAATTCCCCTTATAGTAACGCTCTCTTCTA
PspoVS1-F	AACTGCAGCCATGCGGAGACTACAAGTGA
PspoVS1-R	AACTGCAGTTCCATTCCGCGTTTCCTCCTCGT
PspoVS2-F	AACTGCAGATGAAGATGAATCACAATTGGCG
PspoVS2-R	CGGGATCCTTCCATGTAAGTTGCTCCCTCTATT
EMspoVS1-F	AGTGCAGTGTTAATTGATGTGG
EMspoVS1-R	TTCCATTCCGCGTTTCCTCCTCGT
EMspoVS2-F	GCTGTATGGATGTTATCATTTGGTG
EMspoVS2-R	TTCCATGTAAGTTGCTCCCTCTATT
sigH-F	CGGATCCGGTGGAAGCAGGCTTCGTAAG
sigH-R	GTCGACTGAATTTAAAGTTGTACTTTC
pMAD-F	GGTACCTACGTAGGATCGATCC
pMAD-R	TTGCAGGCCATGCTGTCCA
HDspoVS1-F	GTTAGAAACTGCGGTACAAAAGTG
HDspoVS1-R	CTTTTTAGAGTGTGGGGCGCAGGC
HDspoVS2-F	CGAAACTCTATAATCACCAGCTTG
HDspoVS2-R	CACGAGGCCATACTTCCCTACCAG

### Strain construction.

The putative promoter fragment of P*_spoVS1_* (498 bp) was cloned from B. thuringiensis HD73 genomic DNA using the specific primers PspoVS1-F (with a PstI restriction site) and PspoVS1-R (with a BamHI restriction site). The putative promoter fragment of P*_spoVS2_* (565 bp) was cloned from B. thuringiensis HD73 genomic DNA using the specific primers PspoVS2-F (with a PstI restriction site) and PspoVS2-R (with a BamHI restriction site). The PstI-BamHI fragments of the P*_spoVS1_* promoter and P*_spoVS2_* promoter were then integrated into vector pHT304-18Z containing a promoterless *lacZ* gene (39). The recombinant plasmids P*spoVS1*-*lacZ* and P*spoVS2*-*lacZ* were introduced into the wild-type strain and the *sigH* mutant strain (21). The resulting strains HD(P*spoVS1*-*lacZ*), HD(P*spoVS2*-*lacZ*), HDΔ*sigH*(P*spoVS1*-*lacZ*), and HDΔ*sigH*(P*spoVS2*-*lacZ*) were selected using erythromycin resistance and PCR identification.

All primers for gene deletion were designed according to the B. thuringiensis HD73 genome sequence. The 653-bp fragment upstream from the start codon of *spoVS1* (*spoVS1* fragment A) was amplified using PCR with B. thuringiensis HD73 genomic DNA as the template and spoVS1-a and spoVS1-b as primers. Primers spoVS1-c and spoVS1-d were used to amplify the 679-bp fragment downstream (*spoVS1* fragment B), and primers Km-a and Km-b were used to amplify a 1,473-bp kanamycin resistance gene (*kan*) cassette directed by the PaphA3 promoter from pDG780 (40). *spoVS1* fragment A, *kan*, and *spoVS1* fragment B were ligated together using overlapping PCR with primers SpoVS1-a and SpoVS1-d. The resulting fragment (2,805 bp) was inserted into the BamHI-SalI restriction sites of the erythromycin-resistant, temperature-sensitive plasmid pMAD to generate the pMADΩ*spoVS1*::*km* plasmid. The 642-bp fragment upstream from the start codon of *spoVS2* (*spoVS2* fragment A) was amplified using PCR with B. thuringiensis HD73 genomic DNA as the template and spoVS2-a and spoVS2-b as primers. Primers SpoVS2-c and SpoVS2-d were used to amplify a 606-bp fragment downstream (*spoVS2* fragment B), and primers Km-a and Km-b were used to amplify a 1,473-bp kanamycin resistance gene (*kan*) cassette directed by the PaphA3 promoter from pDG780 (40). *spoVS2* fragment A, *kan*, and *spoVS2* fragment B were ligated together using overlapping PCR with primers SpoVS2-a and SpoVS2-d. The resulting fragment (2,721 bp) was inserted into the BamHI-SalI restriction sites of the erythromycin-resistant, temperature-sensitive plasmid pMAD (41) to generate the pMADΩ*spoVS2*::*km* plasmid. The pMADΩ*spoVS1*::*km* plasmid and pMADΩ*spoVS2*::*km* plasmid were electroporated into B. thuringiensis HD73. Transformants selected on LB agar plates containing erythromycin and kanamycin resistance were identified using PCR with pMAD-F and pMAD-R primers. Gene deletion in the HD73 cells was accomplished using homologous recombination as reported previously (42). The HD(Δ*spoVS1*) mutant was verified using PCR and DNA sequencing with primers HDspoVS1-F and HDspoVS1-R. The HD(Δ*spoVS2*) mutant was verified using PCR and DNA sequencing with primers HDspoVS2-F and HDspoVS2-R.

In addition, *spoVS2* fragment A and *spoVS2* fragment B were ligated together using overlapping PCR with primers SpoVS2-a and SpoVS2-d. The resulting fragment (1,248 bp) was inserted into the BamHI-SalI restriction sites of the erythromycin-resistant, temperature-sensitive plasmid pMAD (41) to generate the pMADΩ*spoVS2* plasmid. The recombinant plasmid was electroporated into the HD(Δ*spoVS1*) strain. Transformants selected on LB agar plates containing erythromycin and kanamycin resistance were identified using PCR with pMAD-F and pMAD-R primers. The *spoVS2* gene deletion in the HD(Δ*spoVS1*) strain was accomplished using homologous recombination as reported previously (42). The double mutant HD(Δ*spoVS1*Δ*spoVS2*) was verified using PCR and DNA sequencing with primers HDspoVS1-F/HDspoVS1-R and HDspoVS2-F/HDspoVS2-R.

To explore the gene function relationship between *spoVS1* and *spoVS2*, a 770-bp fragment containing the *spoVS1* promoter and ORF(RS20190) was amplified using PCR with B. thuringiensis HD73 genomic DNA as the template and spoVS1-F and spoVS1-R as primers. The fragment was linked to the pHT315 shuttle vector (43) using homologous recombination. The recombinant pHTspoVS1 plasmid was then transformed into HD(Δ*spoVS1*) and HD(Δ*spoVS2*) to generate the recombinant strains HD(Δ*spoVS1*Ω*spoVS1*) and HD(Δ*spoVS2*Ω*spoVS1*). A 940-bp fragment containing the *spoVS2* promoter and ORF(RS12225) was amplified using PCR with B. thuringiensis HD73 genomic DNA as the template and spoVS2-F and spoVS2-R as primers. The fragment was linked to the pHT315 shuttle vector (43) using homologous recombination. The recombinant pHTspoVS2 plasmid was then transformed into HD(Δ*spoVS2*) and HD(Δ*spoVS1*) to generate the recombinant strains HD(Δ*spoVS2*Ω*spoVS2*) and HD(Δ*spoVS1*Ω*spoVS2*).

SigH protein with a His tag was purified from E. coli. The expression plasmid pET*sigH* was constructed with PCR amplification of the *sigH* gene from the B. thuringiensis HD73 genome using the primer pair sigH-F (with a BamHI restriction site) and sigH-R (with a SalI restriction site). The DNA fragment was digested with BamHI and SalI, cloned into plasmid pET21b (Novagen, Bloemfontein, South Africa), digested with the same restriction enzymes, and then transferred into E. coli BL21(DE3).

To explore the activities of the *spoIIE*, pro-*sigE*, and pro-*sigK* promoters in the *spoVS* series mutants, a vector containing P*_spoIIE_*-*lacZ*, P*_pro_*_-_*_sigE_*-*lacZ*, or P*_pro-sigK_*-*lacZ* (44) was transformed into the B. thuringiensis wild-type HD73, HD(Δ*spoVS1*), HD(Δ*spoVS2*), and HD(Δ*spoVS1*Δ*spoVS2*) strains.

### Total RNA extraction and RT-PCR analysis.

Total RNA was extracted from B. thuringiensis HD73 cells cultured in SSM medium and harvested at *T*_5_. Reverse transcription-PCR (RT-PCR) identification was performed as described previously (21).

### Determination of transcriptional start sites.

To determine the transcriptional start sites, we employed the SMARTer RACE cDNA amplification kit (Clontech, Mountain View, CA), following the manufacturer’s instructions (35). Gene-specific primers spoVS1RACE-R and spoVS2RACE-R and universal primer mix (UPM) were used to amplify the 5′ ends of *spoVS1* and *spoVS2* mRNAs.

### β-Galactosidase assays.

B. thuringiensis strains containing *lacZ* transcriptional fusions were cultured in SSM medium at 30°C. As the total number of cells gradually increased during the growth of the strain, the sampling volumes were varied by time point (*T*_0_ is the end of the exponential phase, *T*_−_*_n_* is *n* hours before the end of the exponential phase, and *T_n_* is *n* hours after the end of the exponential phase) as follows: 8 ml during the *T*_−2_ period, 4 ml during the *T*_−1_ period, and 2 ml from the *T*_0_ to *T*_8_ period. The cells were harvested and the specific β-galactosidase activities of the samples, expressed as Miller units per milligram of protein, were measured as previously described. The results reported are the mean values from at least three independent trials (45).

### Expression and purification of SigH protein.

The E. coli BL21 strain containing pET*sigH* was grown at 37°C in LB medium supplemented with ampicillin to an optical density at 600 nm (OD_600_) of 0.7. Expression of the SigH-His protein was induced by adding IPTG (isopropyl-β-d-thiogalactopyranoside) to a final concentration of 0.5 mM, and the cultures were incubated for 12 h at 18°C and 150 rpm. The cells were harvested using centrifugation at 13,500 × *g* for 5 min in 50-ml tubes and then resuspended in lysis buffer (50 mM Tris-HCl, pH 8.0, 0.5 M NaCl). Bacteria were lysed on ice using sonication with an ultrasonic cell disruption system. The bacterial lysate was centrifuged at 16,000 × *g* for 10 min at 4°C, with the supernatant containing the solubilized SigH-His protein. The supernatant was filtered through a 0.45-μm membrane filter (Nalgene) and loaded onto a Ni-Sepharose 6 fast-flow column (4 ml) (Pharmacia) previously equilibrated with equilibrium buffer (20 mM Tris-HCl, pH 8.5, 0.5 M NaCl, 50 mM imidazole). The resin with bound protein was washed with five column volumes of equilibrium buffer, and then the target SigH-His protein was eluted with elution buffer (20 mM Tris-HCl, pH 8.5, 0.5 M NaCl, 250 mM imidazole) and collected. Purity was checked using SDS-PAGE followed by Coomassie blue staining. Fractions containing SigH-His protein were desalted using a desalination column and 20 mM Tris-HCl, pH 8.0. The method followed the instructions for the ÄKTA avant 25 protein purification system (46).

### EMSAs.

Using B. thuringiensis HD73 genomic DNA as the template, the DNA fragment with the promoter sequence containing binding sites was amplified using primers marked with FAM (6-carboxyfluorescein) (Table 5). The binding of DNA fragments to protein was determined using electrophoretic mobility shift assays (EMSAs). The 20-μl reaction mixtures contained 0.02 μg or 0.015 μg FAM-labeled DNA, different concentrations of SigH-His protein, binding buffer [10 mM Tris-HCl, pH 7.5, 0.5 mM dithiothreitol (DTT), 50 mM NaCl, 500 ng poly(dI:dC), and 4% glycerol] and was reacted at 25°C for 20 min. The reaction products were detected using electrophoresis in 0.5× Tris-borate-EDTA (TBE) buffer with 5% nondenatured polyacrylamide gel and scanned with a gel imaging system (FLA Imager FLA-5100; Fujifilm).

### Optical microscopy observation.

The HD73, HD(Δ*spoVS1*), HD(Δ*spoVS2*), HD(Δ*spoVS1*Δ*spoVS2*), HD(Δ*spoVS1*Ω*spoVS1*), HD(Δ*spoVS2*Ω*spoVS2*), HD(Δ*spoVS1*Ω*spoVS2*), and HD(Δ*spoVS2*Ω*spoVS1*) strains were cultured in 100 ml SSM medium at 30°C. Samples were collected at designated time points (*T*_24_, *T*_28_, *T*_32_, *T*_48_, and *T*_72_). Cell samples were analyzed with optical microscopy (BX61; Olympus, Tokyo, Japan). Detailed methods were as previously described (44).

### Confocal laser scanning microscopy.

The vital membrane dye FM 4-64 (Molecular Probes, Inc., Eugene, OR, USA) or MitoTracker green FM (ThermoFisher, USA) was dissolved in dimethyl sulfoxide (35). To assess polar septa and engulfment, 1-ml amounts of cells cultured to a designated time point in SSM medium were pelleted and resuspended in 0.02 to 0.05 ml H_2_O. Aliquots (2 μl) of the cell suspensions were placed on slides, stained with FM 4-64 (100 μM) and MitoTracker green FM (MTG; 100 nM) for 1 min (47), and then scanned with a confocal laser scanning microscope (Leica TCS SL; Leica Microsystems, Wetzlar, Germany) (24). Each strain was scanned independently at least three times, and each scan was then viewed in at least five fields. The rate of polar septum formation or incomplete engulfment was defined as the ratio of the number of cells with polar septa (stained with FM 4-64 in the mother cell) or incompletely engulfed cells (stained with MTG) to the total number of cells. The results given are the mean values from at least three independent replicates.

### Sporulation efficiency and quantification of Cry1Ac protein production.

Cells were cultured in a conical flask containing 100 ml of SSM medium at 30°C. One-milliliter samples were taken at *T*_24_, and the total quantities of cells determined. Cell suspensions were heated to 65°C for 20 min to eliminate vegetative cells and then plated onto LB agar medium. The total cell number was defined as the number of colonies at *T*_24_. The spore number was defined as the number of colonies after heat treatment. The sporulation efficiency was defined as the ratio of spore number to total cell number (35).

The HD73, HD(Δ*spoVS1*), HD(Δ*spoVS2*), HD(Δ*spoVS1*Δ*spoVS2*), HD(Δ*spoVS1*Ω*spoVS1*), HD(Δ*spoVS2*Ω*spoVS2*), HD(Δ*spoVS1*Ω*spoVS2*), and HD(Δ*spoVS2*Ω*spoVS1*) strains were grown in SSM medium at 30°C. Cell samples were harvested at *T*_24_, followed by freeze drying until the pellets became lyophilized powders. The same quantities of freeze-dried powder of different strains were dissolved in equal volumes of double-distilled water. Bacterial suspension was disrupted by using a BeadBeater (Biospec Products, Inc., Bartlesville, OK, USA) to make sure all the cells were completely lysed. Twenty-microliter amounts of cell lysates were mixed with 5 μl of 5× loading buffer and boiled for 15 min. Total protein quantitation analyses were performed as previously described (37).
