# Supplementary material for: Identification and Functional Characterization of Two Homologous SpoVS Proteins Involved in Sporulation of Bacillus thuringiensis
Source: Microbiol Spectr. 2021 Oct 6;9(2):e00881-21. doi: 10.1128/Spectrum.00881-21 (PMC8510167; doi:10.1128/Spectrum.00881-21)
Supplement: Supplemental file 1 — Supplemental material. Download SPECTRUM00881-21_Supp_1_seq8.pdf, PDF file, 1.0 MB [file spectrum00881-21_supp_1_seq8.pdf]

# Identification and functional characterization of two homologous SpoVS proteins involved in sporulation of *Bacillus thuringiensis*

Xinlu Liu, Ruibin Zhang, Shuo Hou, Huanhuan Liu, Jiaojiao Wang, Qingyue Yu, Qi Peng, Fuping Song*

State Key Laboratory for Biology of Plant Diseases and Insect Pests, Institute of Plant Protection, Chinese Academy of Agricultural Sciences, Beijing, China

*Corresponding author： Fuping Song

Email: fpsong@ippcaas.cn

X. L. and R. Z. contributed equally to this work. Author order was determined to comply with the requirement for master degree for X. L..

**Running Title**: The role of SpoVS proteins in *Bacillus thuringiensis*

**Keywords:** *Bacillus thuringiensis*, *spoVS*, sporulation, disporic septum, $\sigma^H$

**A**

1 CCATGCGGAGACTACAAGTGAAAAACAAGCGCTAGGTTGGTATGTAGATGGACGTGCTAC

…………………………………………………………………………………………………………

241 TGGCAGAACGCAATTAAGTGCAGTGTTAATTGATGTGGATCCAAATACAGGAAAAGCAAA

301 GAGAATTGAACGTATTTTAATTAATGACGATCAACCATTTTTTGAGTAAATTTCCGTCAA

361 ATTTTAGATAAGTTATTATATTTTAATTTTTTAGAAAAATTACGAAAATTTAGC**AGGA**AT

-35

421 ACGCGCCATTCCTCGT**GAAT**ATAAGTTAAAGT**G**AAATAATTGCTAATACTTTTTAAAGAA

$\sigma^H$ -10 +1

481 ACGAGGAGGAAACGCGGA**ATG**GAA

***RS20190*(*spoVS1*)**

**B**

1 ATGAAGATGAATCACAATTGGCGTGTATGCTTGTACTATAAAAGGAAATAGATTTGGAAT

…………………………………………………………………………………………………………

301 TATTTATTTAGTCTGACAAAACAGAAATATTACCTTTAGCTGTATGGATGTTATCATTTG

361 GTGAAGTTATAATATTTAGCTATATTTATATTAGGTTATTCAGTTTATTTTTTATTTGCG

421 TAGCTGAGC**AGGA**AAAAGTCAGAAAATATA**GAAT**AATATACATAT**G**AAGCATCGTTTGTT

-35 $\sigma^H$ -10 +1

481 TTTGGTGTGAATAGTAATTAAAGATACGCTAAAATATTTAAAATTGTAACGAAAGAGAGA

541 TTAAATAATAGAGGGAGCAACTTAC**ATG**GAA

***RS12225*(*spoVS2*)**

**C**

1 TAACGGAATACAAACCCTTAATGCATTGGTTAAACATTGTAAAGTCTAAAGCATGGATAA

61 TGGGCGAGAAGTAAGTAGATTGTTAACACCCTGGGTCAAAAATTGATATTTAGTAAAATT

121 AGTTGCACTTTGTGCATTTTTTCATAAGATGAGTCATATGTTTTAAATTGTAGTAATGAAA

181 AACAGTATTATATCATAATGAATTGGTATCTTAATAAAAGAGATGGAGGTAACTT**ATG**GA

***cry1Ac***

241 TAACAATCCGAACATCAATGAATGCAT

**D**

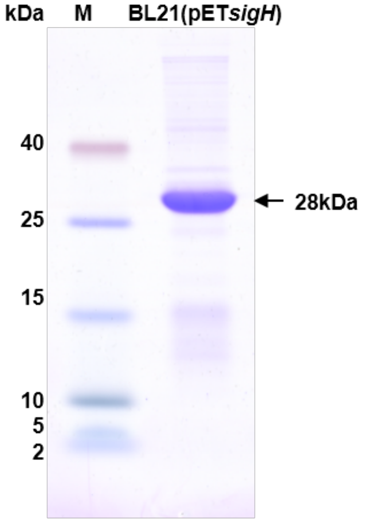

**FIG S1 Sequence analysis of the intergenic region (498 bp) between the *RS20195* and *RS20190* (*spoVS1*) genes of HD73. (A)** The transcription start site (+1) and the putative -35 and -10 motifs are marked. The sequence between the two black arrows is FAM-$P_{spoVS1}$(256bp), the DNA sequence used for EMSA. The green sequence is the primer (Table S2, EMspoVS1-F/R) used to synthesize the FAM-$P_{spoVS1}$ sequence. **(B)** Sequence analysis of the intergenic region (565 bp) between the *RS12220* and *RS12225* (*spoVS2*) genes of *B. thuringiensis* HD73. The transcription start site (+1) and the putative -35 and -10 motifs are marked. The sequence between the two black arrows is FAM-$P_{spoVS2}$(227bp), the DNA sequence used for EMSA. The green sequence is the primer (Table S2, EMspoVS2-F/R) used to synthesize the FAM-$P_{spoVS2}$ sequence. **(C)** The sequence between the two black arrows is FAM-$P_{cry1Ac}$(268bp), the DNA sequence used for EMSA. **(D)** Purification of the SigH-His recombinant protein.

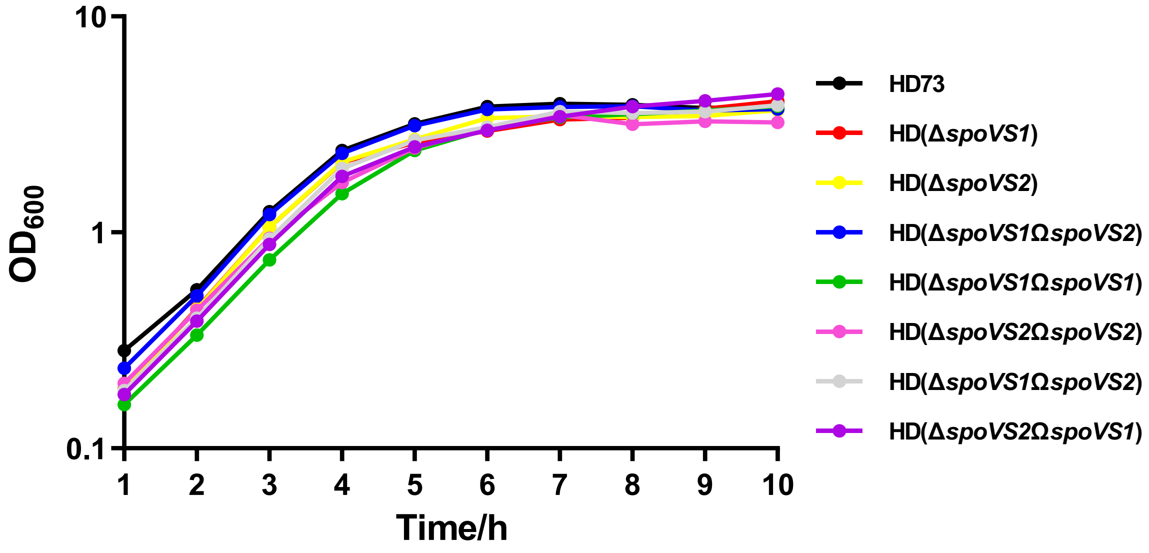


**FIG S2 The effect of *spoVS* gene of deletion and complementation on growth.** The growth curve of HD73 (wild-type strain), HD(Δ*spoVS1*) mutant , HD(Δ*spoVS2*) mutant ,HD(Δ*spoVS1*Δ*spoVS2*) double mutant, HD(Δ*spoVS1*Ω*spoVS1*) strain, HD(Δ*spoVS2*Ω*spoVS2*) strain, HD(Δ*spoVS1*Ω*spoVS2*) strain and HD(Δ*spoVS2*Ω*spoVS1*) strain was measured in SSM at 30°C with shaking at 220 rpm.

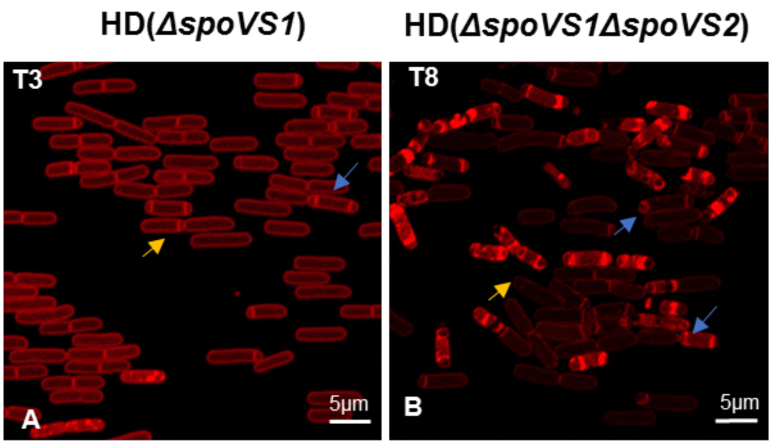


**FIG S3 Observation of Sporulation process by laser scanning confocal microscope.** The polar septum of HD(Δ*spoVS1*) and HD(Δ*spoVS1*Δ*spoVS2*) mutant was observed by laser scanning confocal microscopy at T3 and T8 after incubation in SSM at 30°C with shaking at 220 rpm. Cell membrane is visible as red fluorescence. Red lines represent membranes stained with FM4-64. Yellow arrow indicates a polar septum. Blue arrow indicates disporic septa. Scale Bar, 5μm.

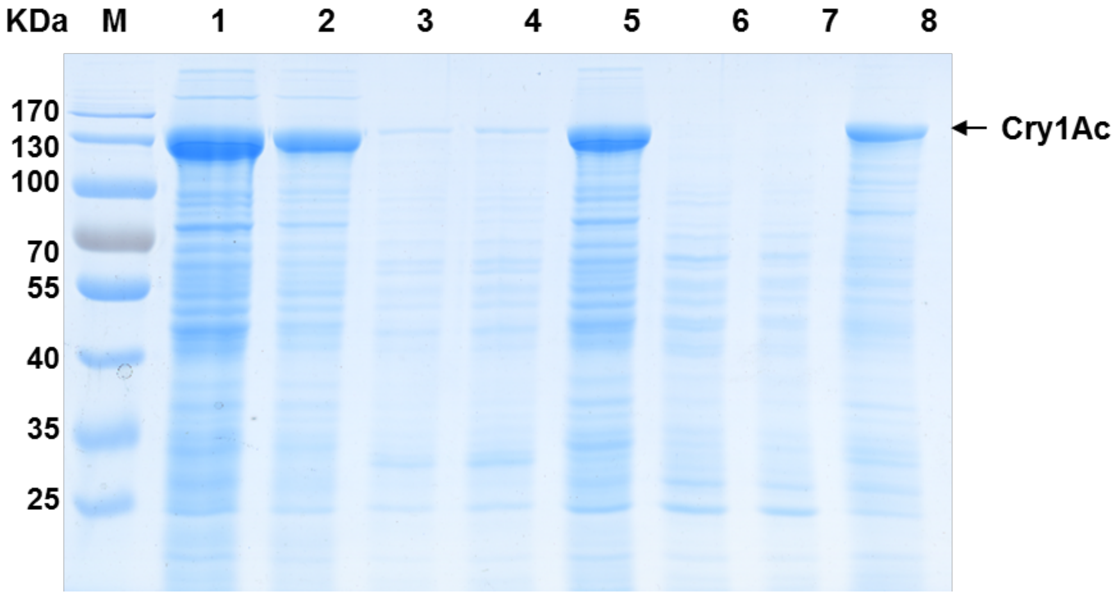


**FIG S4 Cry1Ac expression in the *B. thuringiensis* strain.** SDS-PAGE analysis of Cry1Ac expression in HD73(wild-type strain), HD(Δ*spoVS2*), HD(Δ*spoVS1*), HD(Δ*spoVS1*Δ*spoVS2*), HD(Δ*spoVS2*Ω*spoVS2*), HD(Δ*spoVS1*Ω*spoVS1*), HD(Δ*spoVS2*Ω*spoVS1*) and HD(Δ*spoVS1*Ω*spoVS2*) strain(lane1 to lane 8). Lane M, molecular size markers.

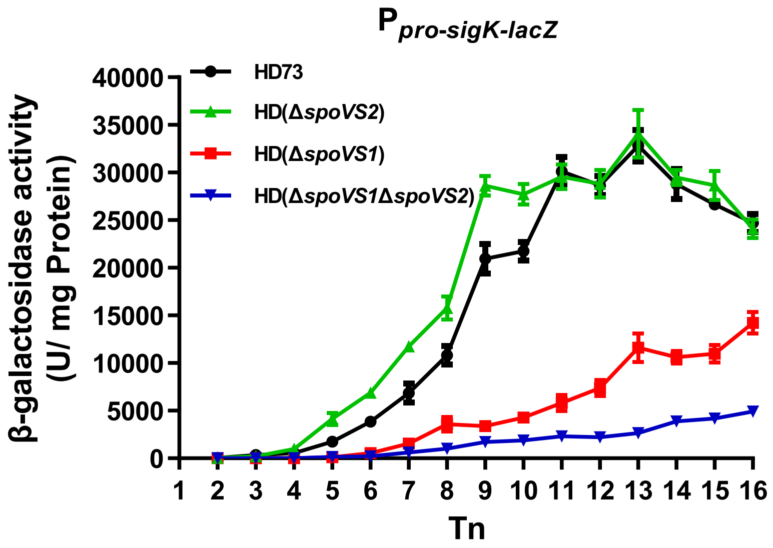


**FIG S5 Effect of various mutations on pro-*sigK* expression.** β-galactosidase activity was assessed for HD73(wild-type), black circle; HD(Δ*spoVS1*) mutant, red square; HD(Δ*spoVS2*) mutant, green triangle; HD(Δ*spoVS1*Δ*spoVS2*) mutant, blue rhombus; containing the plasmid transcriptional fusion $P_{pro-sigK}$-*lacZ*. The bacteria were grown at 30°C in SSM medium and samples were taken at indicated time points. T0 is the end of the exponential growth phase. Tn is n hours after T0. Each value represents the mean of at least three independent replicates. Error bars show the standard error of the mean.

TABLE S1 Distribution of homologous *spoVS* gene in sporulating bacteria

| | Strains | SpoVS | | | | | | | |
|---|---|---|---|---|---|---|---|---|---|
| | | 84AA | 86AA | 87AA | 88AA | 89AA | 90AA | 91AA | Count |
| Bacilli | *Bacillus subtilis subsp.subtilis str.*168 | | 1 | | | | | | 1 |
| | *Bacillus lichenifomis* ATCC 14580 | | 1 | | | | | | 1 |
| | *Bacillus halodurans* C-25 | | 1 | | | | | | 1 |
| | *Bacillus clausii* KSM-K16 | | | 1 | | | | | 1 |
| | *Bacillus cereus* ATCC 10987 | | 1 | | | | | 1 | 2 |
| | *Bacillus anthracis* Ames | | 1 | | | | | 1 | 2 |
| | *Bacillus thuringiensis serovar kurstaki* HD73 | | 1 | | | | | 1 | 2 |
| | *Bacillus toyonensis* | | 1 | | | | | 1 | 2 |
| | *Bacillus mycoides* ATCC 6462 | | 1 | | | | | 1 | 2 |
| | *Bacillus pseudomycoides* 219298 | | 1 | | | | | 1 | 2 |
| | *Bacillus weihenstephanensis* KBAB4 | | 1 | | | | | 1 | 2 |
| | *Bacillus cytotoxicus* | | 1 | | | | | | 1 |
| | *Paenibacillus* sp. JDR-2 | | 1 | | | | | | 1 |
| | *Bacillus megaterium* ATCC 14581 | | 1 | | | | 2 | | 3 |
| Clostridia | *Thermoanaerobacter tengcongensis* MB4 | | 2 | 1 | | | | | 3 |
| | *Moorelia therrnoacetia* ATCC 39073 | | 1 | | | 1 | | | 2 |
| | *Cartoxydothemus hydrogenoformans* Z-2091 | | | | 1 | | | | 1 |
| | *Desulfitobacterium hafniense* Y51 | | 1 | | | | | | 1 |
| | *Clostridium thermocellum* ATCC 27405 | | 1 | | 1 | | | | 2 |
| | *Desulfotomaculum acetoxidans* DSM 771 | | 1 | | | | | | 1 |
| | *Clostridium acetobutylicum* ATCC 824 | | 2 | | | | | | 2 |
| | *Clostridium botulinum* A str. ATCC 3502 | | 1 | | | | | | 1 |
| | *Clostridium perfringens* str. 13 | | 1 | | | | | | 1 |
| | *Clostridium difficile* 630 | | 1 | | | | | | 1 |
| | *Alkaliphilus oremlandii* OhILAs | | 1 | | | | | | 1 |
| | *Symbiobacterium thermophilum* IAM 14863 | 1 | 1 | | | | | | 2 |

TABLE S2 Analysis of amino acid identity in SpoVS of *Bacillus cereus* group

| Strains | Gene code | Amino acid length | Identity (SpoVS2) | Gene code | Amino acid length | Identity (SpoVS1) |
|---|---|---|---|---|---|---|
| ***Bacillus thuringiensis serovar chinensis* CT-43** | **CT43_CH2092** | **91** | **100%** | **CT43_CH3719** | **86** | **100%** |
| ***Bacillus thuringiensis* Bt407** | **BTB_c22060** | **91** | **100%** | **BTB_c38490** | **86** | **100%** |
| ***Bacillus thuringiensis serovar kurstaki* YBT-1520** | **YBT1520_11700** | **91** | **100%** | **YBT1520_20070** | **86** | **100%** |
| ***Bacillus thuringiensis serovar finitimus* YBT-020** | **YBT020_11115** | **91** | **91.20%** | **YBT020_18510** | **86** | **100%** |
| ***Bacillus thuringiensis* YBT-1518** | **YBT1518_11840** | **91** | **100%** | **YBT1518_20735** | **86** | **100%** |
| ***Bacillus thuringiensis serovar konkukian* 97-27** | **BT9727_1977** | **91** | **90.10%** | **BT9727_3517** | **86** | **100%** |
| ***Bacillus cereus* B4264** | **BCB4264_A2160** | **91** | **100%** | **BCB4264_A3875** | **86** | **100%** |
| ***Bacillus cereus* G9842** | **BCG9842_B3154** | **91** | **100%** | **BCG9842_B1424** | **86** | **100%** |
| ***Bacillus cereus* ATCC 14579** | **BC2142** | **91** | **98.9%** | **BC3776** | **86** | **100%** |
| ***Bacillus cereus* ATCC 10987** | **BCE_2222** | **90** | **92.20%** | **BCE_3811** | **86** | **100%** |
| ***Bacillus cereus* FRI-35** | **BCK_23855** | **90** | **92.20%** | **BCK_16360** | **86** | **100%** |
| ***Bacillus anthracis* CDC 684** | **BAMEG_2438** | **91** | **91.20%** | **BAMEG_0719** | **86** | **100%** |
| ***Bacillus anthracis* A0248** | **BAA_2219** | **91** | **91.20%** | **BAA_3937** | **86** | **100%** |
| ***Bacillus anthracis* Ames** | **BA_2154** | **91** | **91.20%** | **BA_3912** | **86** | **100%** |
| ***Bacillus anthracis* A16** | **A16_21900** | **91** | **91.20%** | **A16_39200** | **86** | **100%** |
| ***Bacillus anthracis* Vollum** | **DJ46_955** | **91** | **91.20%** | **DJ46_2615** | **86** | **100%** |
| ***Bacillus toyonensis*** | **Btoyo_4728** | **91** | **91.20%** | **Btoyo_1011** | **86** | **100%** |
| ***Bacillus mycoides* ATCC 6462** | **BG05_3849** | **91** | **85.70%** | **BG05_2247** | **86** | **100%** |
| ***Bacillus mycoides* WSBC 10204** | **Bwei_2877** | **91** | **85.70%** | **Bwei_1181** | **86** | **100%** |
| ***Bacillus weihenstephanensis* KBAB4** | **BcerKBAB4_1997** | **91** | **85.70%** | **BcerKBAB4_3547** | **86** | **100%** |
| ***Bacillus pseudomycoides* 219298** | **DJ92_2039** | **91** | **78.00%** | **DJ92_819** | **86** | **100%** |
